# Serrated lithium fluoride nanofibers-woven interlayer enables uniform lithium deposition for lithium-metal batteries

**DOI:** 10.1093/nsr/nwac183

**Published:** 2022-09-01

**Authors:** Shuangshuang Tan, Yalong Jiang, Shuyan Ni, Hao Wang, Fangyu Xiong, Lianmeng Cui, Xuelei Pan, Chen Tang, Yaoguang Rong, Qinyou An, Liqiang Mai

**Affiliations:** State Key Laboratory of Advanced Technology for Materials Synthesis and Processing, Wuhan University of Technology, Wuhan 430070, China; College of Materials Science and Engineering, Chongqing University, Chongqing 400030, China; State Key Laboratory of Advanced Technology for Materials Synthesis and Processing, Wuhan University of Technology, Wuhan 430070, China; State Key Laboratory of Advanced Technology for Materials Synthesis and Processing, Wuhan University of Technology, Wuhan 430070, China; State Key Laboratory of Advanced Technology for Materials Synthesis and Processing, Wuhan University of Technology, Wuhan 430070, China; State Key Laboratory of Advanced Technology for Materials Synthesis and Processing, Wuhan University of Technology, Wuhan 430070, China; State Key Laboratory of Advanced Technology for Materials Synthesis and Processing, Wuhan University of Technology, Wuhan 430070, China; State Key Laboratory of Advanced Technology for Materials Synthesis and Processing, Wuhan University of Technology, Wuhan 430070, China; State Key Laboratory of Advanced Technology for Materials Synthesis and Processing, Wuhan University of Technology, Wuhan 430070, China; Wuhan National Laboratory for Optoelectronics, Huazhong University of Science and Technology, Wuhan 430074, China; State Key Laboratory of Advanced Technology for Materials Synthesis and Processing, Wuhan University of Technology, Wuhan 430070, China; Foshan Xianhu Laboratory of the Advanced Energy Science and Technology Guangdong Laboratory, Foshan 528200, China; State Key Laboratory of Advanced Technology for Materials Synthesis and Processing, Wuhan University of Technology, Wuhan 430070, China; Foshan Xianhu Laboratory of the Advanced Energy Science and Technology Guangdong Laboratory, Foshan 528200, China

**Keywords:** lithium fluoride, serrated nanofibers, LiF interlayer, uniform Li deposition, lithium-metal batteries

## Abstract

The uncontrollable formation of Li dendrites has become the biggest obstacle to the practical application of Li-metal anodes in high-energy rechargeable Li batteries. Herein, a unique LiF interlayer woven by millimeter-level, single-crystal and serrated LiF nanofibers (NFs) was designed to enable dendrite-free and highly efficient Li-metal deposition. This high-conductivity LiF interlayer can increase the Li^+^ transference number and induce the formation of ‘LiF–NFs-rich’ solid–electrolyte interface (SEI). In the ‘LiF–NFs-rich’ SEI, the ultra-long LiF nanofibers provide a continuously interfacial Li^+^ transport path. Moreover, the formed Li–LiF interface between Li-metal and SEI film renders low Li nucleation and high Li^+^ migration energy barriers, leading to uniform Li plating and stripping processes. As a result, steady charge–discharge in a Li//Li symmetrical cell for 1600 h under 4 mAh cm^−2^ and 400 stable cycles under a high area capacity of 5.65 mAh cm^−2^ in a high-loading Li//rGO–S cell at 17.9 mA cm^−2^ could be achieved. The free-standing LiF–NFs interlayer exhibits superior advantages for commercial Li batteries and displays significant potential for expanding the applications in solid Li batteries.

## Teaser

A unique lithium fluoride interlayer woven by millimeter-level, single-crystal and serrated nanofibers enables uniform Li metal plating/stripping behaviors, effectively promoting the application of high-energy and high-safety Li metal batteries.

## INTRODUCTION

High-energy and high-safety batteries play a crucial factor in promoting the development of electrical vehicles, unmanned aerial vehicles and other strategic emerging industries [1–5]. Lithium-metal batteries (LMBs), such as lithium–sulfur (Li–S) batteries, lithium–LiNi*_x_*Mn*_y_*Co_1−_*_x_*_−_*_y_*O_2_ (Li–NCM) batteries and lithium–oxygen batteries are considered as highly promising candidates for next-generation high-energy energy-storage systems, owing to the ultra-high theoretical capacity (3860 mAh g^−1^) and lowest electrochemical potential (−3.04 V vs standard hydrogen electrode) of lithium-metal anodes [6,7]. However, the uncontrollable growth of lithium dendrites and large overpotential of lithium deposition/dissolution still lead to the poor cycling life and rate performance of LMBs [8–10]. More importantly, lithium dendrites easily lead to accidental short circuits, failure and thermal runaway of cells, further resulting in fires and even explosions [11,12]. They drastically impede the commercial applications of LMBs.

In order to limit the growth of lithium dendrites and reduce the overpotential of lithium deposition/dissolution, researchers have proposed various modification strategies, such as electrolyte composition modification [13,14], artificial solid–electrolyte interface (SEI) design [15–19], the utilization of solid electrolytes [20–24], separator modification [25–27], the introduction of a 3D conductive/lithophilic lithium host [28,29] and so forth [30]. By contrast, introducing the modified interlayer between separator and Li anode is a facile and low-cost method in commercial cell assembly for suppressing the growth of lithium dendrites [31–33]. It can avoid the complex chemical reaction on the active Li-metal surface and the interference of water and oxygen. The current work has mainly focused on designing organic polymers or lithophilic metal compound interlayers that could absorb Li^+^ ions through functional groups or polar bonds to adjust the Li^+^ concentration on the Li surface, further tuning Li plating/stripping [34–36]. However, their low Li^+^ conductivity would lead to a low Li^+^ transference number [33]. In comparison, Li^+^-ion conductor materials, such as Li_2_S, Li_2_O, Li_2_CO_3_, Li_3_N, LiF, Li_3_PS_4_, Li_1+_*_x_*Al*_x_*Ge_2–_*_x_*(PO_4_)_3_ and so forth, are expected to both control the Li^+^ ion flux and decrease the Li deposition resistance on Li-metal. These materials have been employed as key components in artificial SEI layers or solid electrolytes [37–41]. Among them, LiF material possesses good chemical stability in the air, high Li^+^ conductivity and light weight, so it is considered an ideal component for the separator interlayer [42]. Density functional theory (DFT) calculations have also demonstrated that lithium halides have lower Li^+^ diffusion energy barriers (0.03–0.16 eV) compared with Li_2_S, Li_2_O and Li_2_CO_3_ [13,22]. However, the as-reported all-inorganic LiF SEI film synthesized by chemical fluorination on Li-metal is dense. It is prone to rupturing under rapid and large volume change (dozens of micrometers) during high-rate charge–discharge [23,43]. Besides, cubic LiF has an inherent propensity to grow into a cuboid particle, which is difficult to process into a free-standing membrane as an interlayer [44]. Therefore, designing and preparing a free-standing and porous LiF interlayer is difficult and has barely been reported. More importantly, a free-standing LiF interlayer has large potential for compatibility with various liquid or solid LMBs under industrial conditions.

Herein, millimeter-level, single-crystal and serrated LiF nanofibers (LiF–NFs) were synthesized using a facile ‘ice-sublimation’-induced crystallization process. The growth orientations of the sawtooth and trunk of serrated LiF–NFs were revealed in detail using microscope observations and their growth mechanisms were further discussed. When prepared as a separator interlayer (IL) by suction filtration, its thermostability, wettability in electrolyte and Li^+^ transference number were systematically evaluated. In addition, *in situ* optical microscope technology demonstrated uniform lithium deposition behavior when covered by a LiF–NFs interlayer (LiF–NFs-IL). Consequently, a symmetrical Li//Li cell with LiF–NFs-IL delivers a low polarization voltage and excellent cycling stability at different current densities. Moreover, when introduced into high-loading LMBs, LiF–NFs-IL gives high area-specific capacity and excellent rate and cycling performances.

## RESULTS AND DISCUSSION

**Figure 1.**
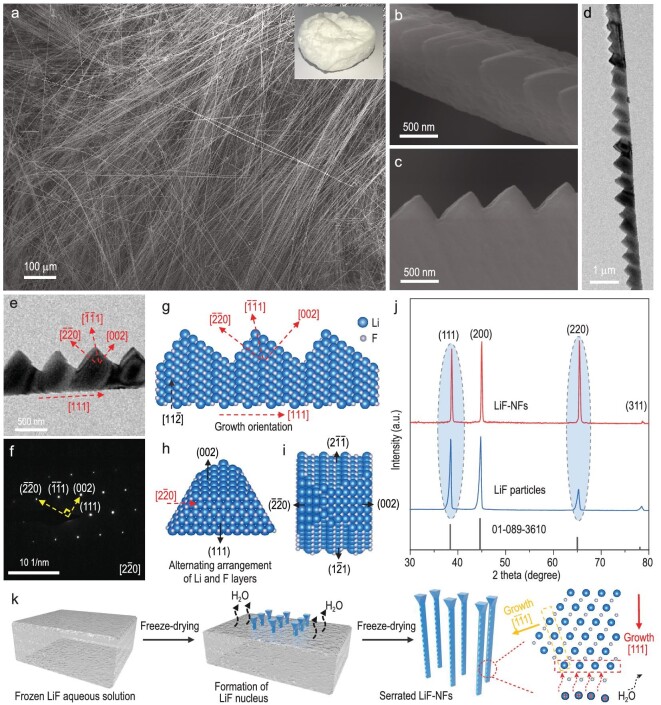
Growth mechanism and structural characterization. (a–c) SEM images of ultra-long serrated LiF–NFs; inset is the corresponding optical image. (d and e) TEM images and (f) SAED pattern of serrated LiF–NF. (g) Atomic model of serrated LiF–NF enclosed with ($\bar{2}\bar{2}$0), (002), (2$\bar{1}\bar{1}$) and (1$\bar{2}$1) facets, and the projections of this NF along (h) [111] and (i) [11$\bar{2}$] zone axis, respectively. (j) XRD patterns of LiF–NFs and LiF particles. (k) Schematic illustration of the growth process of ultra-long serrated LiF–NFs.

The optical image of the as-prepared LiF–NFs using the freeze-drying method is shown in the inset of Fig. 1a. Unlike commercial LiF powder, the as-prepared LiF–NFs is a white cotton-like aerogel. Scanning electron microscope (SEM) imaging showed that it is made of an ultra-long LiF nanofiber structure with a length of a few millimeters (Fig. 1a). In the enlarged SEM images, ultra-long LiF–NFs primarily exhibit uniquely serrated morphology (Fig. 1b and c, and Supplementary Fig. S1). In contrast, for the routine solution-drying method, only micron-sized cubic LiF particles were obtained (Supplementary Fig. S2). Transmission electron microscopy (TEM) images indicate that there are no pores or cracks in single serrated LiF–NF (Fig. 1d and e), in which the sawtooth arrangement is ordered. The corresponding selected area electron diffraction (SAED) pattern further demonstrates the single-crystal feature of the serrated LiF–NF, while its growth orientation is analysed in the [111] direction (Fig. 1f). Two sides of a single sawtooth are parallel to (220) and (002) planes, respectively. A speculated serrated nanofiber structure with exposed ($\bar{2}\bar{2}$0), (002), (2$\bar{1}\bar{1}$) and (1$\bar{2}$1) facets was constructed as shown in Fig. 1g–i. It accurately shows the arrangements of Li and F atoms in a single serrated LiF–NF. LiF is a typical face-centered cubic structure with Fm-3m space group. The {100} planes are the most stable facets of LiF, which is consistent with NaCl crystals [45]. The <111> orientations are the fastest growth directions of LiF [46], which may be ascribed to the fact that the alternately arranged Li and F layers along with the <111> orientations produce strong electrostatic forces for the followed ion adsorptions during the growth process of LiF–NF. Consequently, the growth orientations of the sawtooth and trunk of serrated LiF–NF are [$\bar{1}\bar{1}1$] and [111] directions, respectively. The X-ray diffraction (XRD) patterns of LiF–NFs and LiF particles further verify the growth orientations of <111> and a high percentage of (110) arrangements (Fig. 1j).

To get deeper sight into the growth mechanism of serrated LiF–NFs assisted by ice sublimation (Fig. 1k), a synthetic process from the frozen aqueous LiF solution to white aerogel is shown in Supplementary Fig. S3. When the frozen solution was quickly transferred into a low-pressure freeze dryer, the white LiF crystal nuclei would form on the surface of the frozen solution. The continuous ice sublimation leads to the vertical growth of LiF–NFs. A thin LiF–NFs film with a thickness of ∼2 mm was further prepared to observe the crystal nuclei on top and the growth behavior of LiF–NFs (Supplementary Fig. S4). It is found that the LiF–NFs vertically grow in clusters, while the crystal nuclei on top are square-shaped. These results suggest that the growth orientation of LiF–NFs is the result of competition among growth along with different crystal directions. Thus, that is dominated by the fast growth of {111} facets. Interestingly, it is also found that several LiF–NFs contain branches at an angle of 110^o^ to the trunk (Supplementary Fig. S5a). The same SAED patterns of trunk and branch demonstrate the single-crystal feature (Supplementary Fig. S5b–d). Besides, the growth orientation of the branch could be identified as [$\bar{1}\bar{1}1$], which is consistent with that of the sawtooth of serrated LiF–NF. It indicates that the branch grew from the sawtooth along the [$\bar{1}\bar{1}1$] direction during ice sublimation. It further suggests that the lengths of the branch and sawtooth are determined by the ice-sublimation rate that is controlled by the vacuum degree. A high vacuum degree would lead to fast ice sublimation and the ordered formation of the LiF–NF sawtooth. Conversely, a poor vacuum degree would cause the over-growth of the LiF–NF branch. Controlling the vacuum degree during the synthetic process can tune the morphology of LiF–NFs.

**Figure 2.**
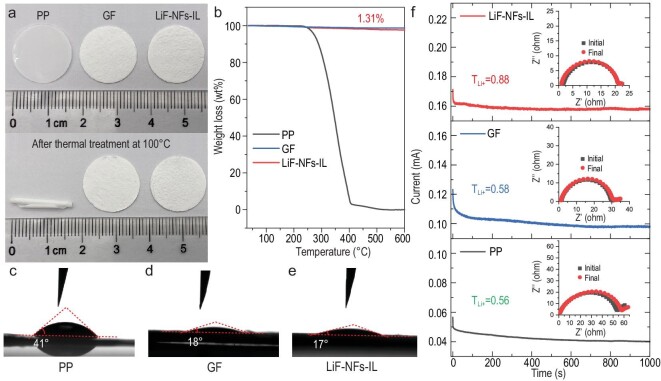
Thermal stability, wettability and ion transference number. (a) Optical photos of PP, GF and LiF–NFs-IL before and after thermal treatment at 100°C for 1 h. (b) TG curves of PP, GF and LiF–NFs-IL. The contact angles of ether-based LiTFSI electrolyte on (c) PP, (d) GF and (e) LiF–NFs-IL. (f) The *i–t* curves of Li//Li symmetrical cells using PP, GF and LiF–NFs-IL; the insets are the corresponding EIS profiles before and after CA test.

A 1D LiF nanostructure with such serrated and single-crystal features has not been achieved previously, to the best of our knowledge. This structure provides an ultra-long and continuous Li^+^-ion transport path for tuning the ionic field on the Li-metal surface. A LiF–NFs film compressed by 5 MPa and the stainless steel (SS) sheets were assembled into a symmetrical SS//LiF//SS cell. The ionic conductivity of the LiF–NFs film was calculated as ∼8.78 × 10^−5^ S cm^−1^, according to the electrochemical impedance spectroscopy (EIS) profile at 25^o^C (Supplementary Fig. S6). The high ionic conductivity substantially exceeds the LiF films prepared by chemical fluorination on Li-metal in the literature (∼10^−9^ S cm^−1^) [37,47]. The ultra-long LiF–NFs exhibit shows great potential in LMB interlayers, artificial SEI and solid electrolytes. Therefore, a porous LiF–NFs-IL was further prepared using the simple suction filtration method (Supplementary Fig. S7). The thickness of the porous LiF–NFs-IL is ∼200 μm (Supplementary Fig. S8). It can be easily transferred from a glass-fiber (GF) membrane. The thermal stabilities of the separator or interlayer are important to avoid decomposition when thermal runaway occurs in LMBs. The optical pictures of polypropylene (PP), GF separators and LiF–NFs-IL before and after thermal treatment at 100°C are shown in Fig. 2a. After being treated for 1 h, the PP separator was rolled up, while the GF and LiF–NFs-IL were unchanged in color and shape. Thermogravimetry (TG) curves of the three membranes further demonstrate the fast thermal degradation of PP at ∼250^o^C and the excellent thermal stability of LiF–NFs-IL up to 600^o^C (Fig. 2b). The burning tests also demonstrate that LiF–NFs-IL shows excellent flame resistance (Supplementary Fig. S9). In addition, the small contact angle (17^o^) indicates the better wettability of LiF–NFs-IL for ether-based lithium bis(trifluoromethyl sulfonyl)imide (LiTFSI) electrolyte (1 M LiTFSI in equal dimethoxyethane/1,3-dioxolane (DME/DOL)) compared with those of PP (41^o^) and GF (18^o^) separators (Fig. 2c–e). The Li^+^ ion transference number (${T}_{L{i}^ + }$) is also an essential factor for evaluating the Li^+^ migration property in the separator and electrolyte. The Li//Li symmetrical cells with different PP, GF and LiF–NFs-IL were assembled to evaluate the ${T}_{L{i}^ + }$ combined by EIS before and after chronoamperometry (CA) test and calculated using the following equation [48,49]:

$$\begin{equation*}
{T}_{L{i}^ + } = {I}\!_s\left( {\Delta V - {I}_0{R}_0} \right)/{I}_0\left( {\Delta V - {I}_s{R}_s} \right),
\end{equation*}$$

where *I*_0_ and *R*_0_ are the initial current and interfacial resistance (*R_ct_*), respectively. *ΔV* is the constant applied voltage (10 mV), *I_s_* and *R_s_* are the steady-state current and *R_ct_*, respectively. The initial *R_ct_* of the Li//LiF–NFs-IL//Li cell (19 Ω) is significantly lower than that of the Li//GF//Li (28.5 Ω) and Li//PP//Li (51 Ω) cells, which suggests that contact between the LiF–NFs and Li-metal would reduce the interfacial Li^+^ ion transference resistance (Fig. 2f). After the CA tests, the ${T}_{L{i}^ + }$ of the LiF–NFs-IL could be calculated as 0.88, which is higher than the 0.58 of GF and 0.56 of PP. It may be attributed to the high interfacial ionic diffusion rate between the single-crystal LiF–NFs and the electrolyte.

**Figure 3.**
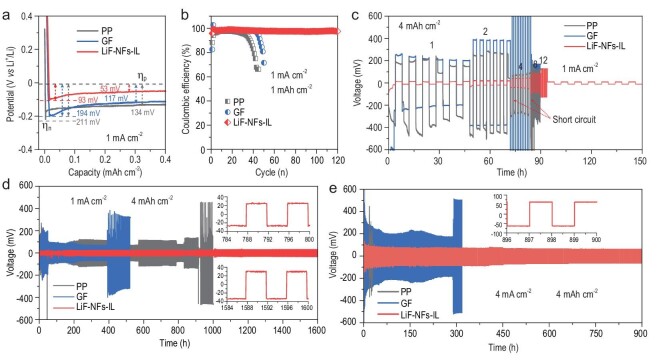
Electrochemical performances of Li half cells. (a) The first discharge profiles of Li//Cu asymmetrical cells with PP, GF and LiF–NFs-IL at 1 mA cm^−2^. (b) CE and cycling stabilities of three kinds of Li//Cu cells. (c) Rate performances of Li//Li symmetrical cells with PP, GF and LiF–NFs-IL at 1, 2, 4, 8 and 12 mA cm^−2^ with an area capacity of 4 mAh cm^−2^. Cycling stabilities of three kinds of Li//Li cells at (d) 1 mA cm^−2^ and (e) 4 mA cm^−2^ with 4 mAh cm^−2^.

Asymmetrical Li//Cu batteries using different PP, GF and LiF–NFs-IL were assembled to investigate the overpotential and coulombic efficiency (CE) of Li deposition. The used electrolyte is the 1 M LiTFSI in equal DME/DOL. The Li//Cu battery with LiF–NFs-IL shows lower overpotentials of Li nucleation (*η*_n_, 93 mV) and growth (*η*_p_, 53 mV) compared with those of Li//Cu batteries with GF (194, 117 mV) and PP (211, 134 mV) separators (Fig. 3a) [50,51]. This indicates that the LiF–NFs-IL can dramatically reduce the Li^+^ nucleation barrier on the electrode surface. The CE and cycling stability of three cells are shown in Fig. 3b. Under the protocol of 1 mA cm^−2^ and 1 mAh cm^−2^, the Li//Cu cell with LiF–NFs-IL exhibits an initial CE of 95.8%, which remains at ∼97.5% after 120 stable cycles. By contrast, the Li//Cu cells using PP and GF separators only deliver a low initial CE of <90% and poor cycling stabilities. When the area capacity is increased to 4 mAh cm^−2^, the Li//Cu cell with LiF–NFs-IL still displays a stable CE of 96% after 60 cycles (Supplementary Fig. S10). These results indicate that introducing LiF–NFs-IL can effectively reduce the potential polarization and suppress the formation of lithium dendrite, thus increasing the CE and cycling stability of the Li-metal anode.

The protection effect of LiF–NFs-IL for the Li anode was also evaluated based on Li//Li symmetrical cells. LiF–NFs-IL renders excellent reversibility of Li plating and stripping at different current densities of 1, 2, 4, 8 and ≤12 mA cm^−2^ with 4 mAh cm^−2^ (Fig. 3c). The corresponding overpotentials are 15, 22, 43, 85 and 125 mV, respectively. Meanwhile, the behavior of a short circuit did not occur at high current densities of 12 mA cm^−2^. After undergoing high-rate Li plating/stripping, the Li//Li cell with LiF–NFs-IL can still stably cycle with a voltage hysteresis of 11 mV at 1 mA cm^−2^. By comparison, the Li//PP//Li and Li//GF//Li cells exhibit tremendous overpotentials of >200 mV at 1 mA cm^−2^. When the current density is increased to 4 and 8 mA cm^−2^, the Li//PP//Li and Li//GF//Li cells suffer from short circuits, respectively. This also indicates that a high current density will accelerate the growth of lithium dendrite owing to uneven electric and ionic fields, leading to a rapid short circuit of Li-metal batteries. The cycling stabilities of Li//Li symmetrical cells at 1 mA cm^−2^ with 4 mAh cm^−2^ are shown in Fig. 3d. After cycling for 900 and 400 h, the Li//PP//Li and Li//GF//Li cells display drastically increased overpotentials of 460 and 353 mV, respectively. This indicates that structural failure of the Li anode and exhaustion of the electrolyte have occurred, owing to continuous formations of dendrite and ‘dead lithium’ [52]. However, the Li//Li cell with LiF–NFs-IL can stably cycle with a slow increase in overpotential from 20 to 30 mV after 1600 h. Moreover, the steady and flat voltage profiles at 800 and 1600 h (insets of Fig. 3d) further demonstrate fast Li^+^ transport and uniform Li-metal deposition/dissolution [10]. When the current density is increased to 4 mA cm^−2^, the Li//PP//Li cell suffers from rapid electrode failure and short circuit, which is consistent with the rate measurement. The Li//GF//Li cell exhibits better stability near 300 h. By contrast, the Li//Li cell with LiF–NFs-IL still delivers a long cycling life of >900 h with a low overpotential of 63 mV. These results demonstrate that LiF–NFs-IL actually improves the Li deposition behavior and suppresses the formation of lithium dendrites.

In order to demonstrate the versatility of LiF–NFs-IL, the LiF–NFs-IL//PP//LiF–NFs-IL composite separator was also employed in the Li//Li symmetrical cell (Supplementary Fig. S11). At 1 mAh cm^−2^ with 4 mAh cm^−2^, the cell exhibits excellent cycling stability (1600 h) and low overpotential (∼30 mV). The distribution density of LiF–NFs and the thickness of LiF–NFs-IL may affect the performance and deposition morphology of Li-metal. The 200-μm LiF–NFs-IL was pressed using a high pressure of 3.5 MPa to obtain the thinner interlayer with a thickness of ∼100 μm, while its surface distribution density was effectively enhanced (Supplementary Fig. S12). A symmetrical Li//Li cell with the pressed LiF–NFs-IL exhibits excellent cycling stability of 1200 h and low overpotential of ∼20 mV at 1 mA cm^−2^ with 1 mAh cm^−2^. A thinner LiF–NFs-IL with a thickness of ∼40 μm was further prepared on a GF separator (Supplementary Fig. S13). The corresponding Li//Cu cell exhibits a CE of 97.0% after 100 stable cycles at 1 mA cm^−2^ with 1 mAh cm^−2^. The Li//Li cell also displays excellent cycling stability (1200 h) and low overpotential (∼20 mV). These results indicate that the 40-μm LiF–NFs-IL could still effectively reduce the potential polarization and suppress the formation of lithium dendrite. It is expected to be processed into a thin high-strength membrane for meeting commercialization requirements.

**Figure 4.**
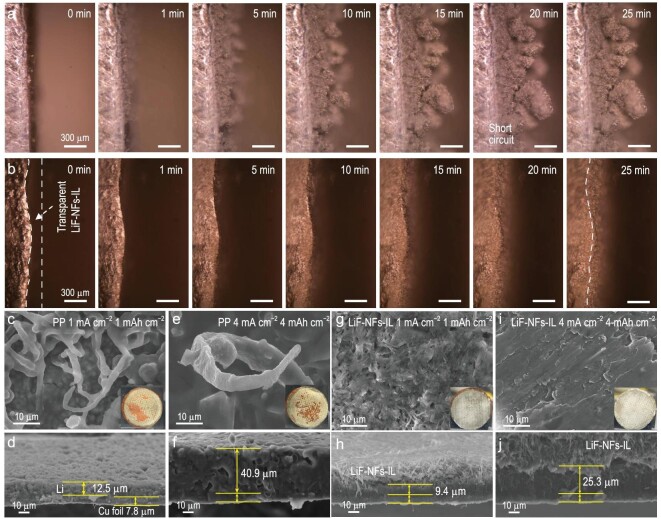
*In situ* optical observation and Li deposition morphology. The recorded images at different times of Li deposition on (a) bare Li-metal surface and (b) Li–LiF interface based on *in situ* optical microscopy technology. The frontal and cross-section SEM images of Li deposition on the Cu foil at 1 mA cm^−2^ with 1 mAh cm^−2^ and 4 mA cm^−2^ with 4 mAh cm^−2^, when using (c–f) PP and (g–j) LiF–NFs-IL.

*In situ* optical microscopy technology was carried out to investigate the Li deposition morphology on Li-metal with and without LiF–NFs-IL. The *insitu* cells were subjected to a high current density of 4 mA cm^−2^ (based on the 1 × 1 cm^2^ Li foil). The recorded images at different times are displayed in Fig. 4. On the deposition side of the Li//Li symmetrical cell, plenty of Li whiskers on the bare Li-metal surface immediately appeared after 1 min of discharge (Fig. 4a and Supplementary Movie S1). Then the Li whiskers rapidly grew into cluster-like dendrites as time went on. At 20 min, the Li//Li cell was short-circuited, after which the fluffy lithium dendrites stopped growing. In comparison, a dense and flat Li-metal layer can be uniformly deposited on a Li-metal surface when covered by LiF–NFs-IL (Fig. 4b and Supplementary Movie S2). It is noteworthy that the LiF–NFs-IL after electrolyte infiltration was transparent (Supplementary Fig. S14). These observations distinctly demonstrate that the LiF–NFs-IL can effectively suppress the formation of Li dendrite at a high current density.

The practical morphologies of Li deposition on Cu foil in asymmetrical Li//Cu batteries using PP, GF and LiF–NFs-IL were verified using SEM measurement. At 1 mA cm^−2^ with 1 mAh cm^−2^, the optical photo shows the obviously uneven Li deposition on the Cu foil in the Li//PP//Cu cell (inset of Fig. 4c). Meanwhile, the SEM image shows a large number of Li dendrites with a diameter of ∼2 μm (Fig. 4c). The deposition thickness of the fluffy and porous Li layer made of whisker-like Li dendrites is 12.5 μm (Fig. 4d). When the current density and area capacity are increased to 4 mA cm^−2^ and 4 mAh cm^−2^, respectively, the diameter of the Li dendrite can reach ∼10 μm accompanied by an increased length (Fig. 4e). The thickness of the porous Li deposition layer can reach 40.9 μm (Fig. 4f), thus its actual density can be calculated as ∼0.253 g cm^−3^, which is much lower than the theoretical density of Li-metal (0.534 g cm^−3^). A large number of pores is not conducive to electronic conduction during the stripping process, further leading to the formation of ‘dead lithium’ and reducing the CE. In the case of using a GF separator, vertical Li dendrites with similar diameters are still observed after depositing for 1 and 4 mAh cm^−2^ (Supplementary Fig. S15a and c). The corresponding thicknesses of the Li deposition layers are 11.9 and 36.7 μm, respectively (Supplementary Fig. S15b and d). In contrast, after introducing the LiF–NFs-IL, the deposited Li-metal is uniform and smooth, and it is stuck with a thin LiF–NFs-IL (Fig. 4g). The thickness of the dense Li deposition layer is 9.4 μm (Fig. 4h). The plated Li-metal is mainly in the interface between the Cu and the LiF–NFs-IL. Under the protocol of 4 mA cm^−2^ and 4 mAh cm^−2^, the flat and dense Li deposition layer with a thickness of 25.3 μm can be measured (Fig. 4i and j). The calculated density is 0.41 g cm^−3^, which is much higher than that of using the PP separator, and is close to the theoretical value. Moreover, after cycling for 100 h, a mass of dendrites and ‘dead lithium’ could be observed in the Li-metal anodes of the symmetrical Li//Li cells with PP and GF separators (Supplementary Fig. S16a and b). By comparison, the Li-metal coated with LiF–NFs-IL remains a flat and dense surface after the cycling process (Supplementary Fig. S16c). These results show that the introduction of LiF–NFs-IL can homogenize lithium deposition well and avoid the formation of lithium dendrites or ‘dead lithium’.

**Figure 5.**
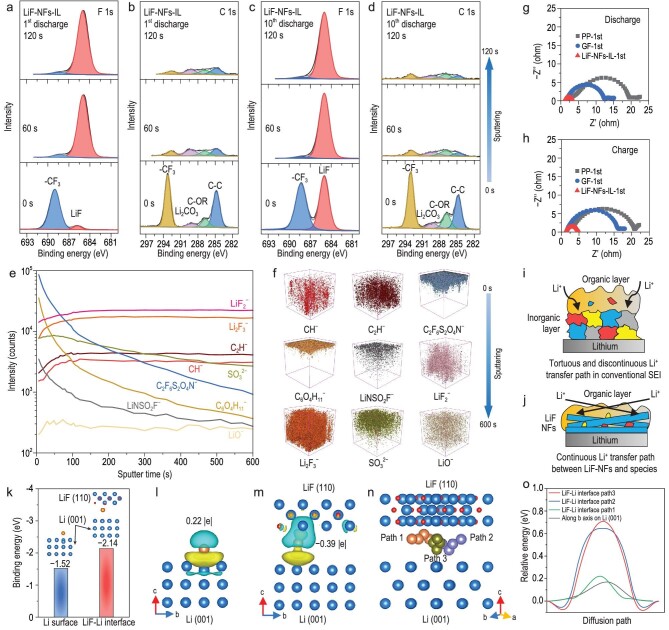
SEI analysis and theoretical calculation. XPS F1s (a and c) and C1s (b and d) depth spectra of SEI film on Li-metal in a Li//Cu cell using LiF–NFs-IL after 1st and 10th discharge at 1 mA cm^−2^ with 1 mAh cm^−2^ (the XPS intensity of each element signal was autoscaled while each column has the same intensity scale). (e) TOF–SIMS depth profiles and (f) 3D rendering models of SEI film when using LiF–NFs-IL after 10th discharge at 1 mA cm^−2^ with 1 mAh cm^−2^. EIS plots of the Li//Li cells after (g) discharging and (h) charging for 1 h at 1 mA cm^−2^. Schematic illustrations of the (i) common SEI and (j) ‘LiF–NFs-rich’ SEI films. The binding energies (k), adsorption configurations and charge density difference plots (l and m) of Li adatoms on the Li (001) facet and in the Li–LiF interface. (n) Diffusion paths of Li atoms in the Li–LiF interface along the *b*-axis (Path 1), *a*-axis (Path 2) and [110] direction (Path 3). (o) The relative energy profiles of Li atom diffusion in four cases.

The components of the SEI film play a significant role in its stability and ionic conductivity, further determining the deposition morphology of Li-metal, the consumption amount of the electrolyte and the CE of LMBs. The introduction of LiF–NFs-IL may change the components and structure of the SEI film. Therefore, X-ray photoelectron spectroscopy (XPS) and time-of-flight secondary ion mass spectrometry (TOF–SIMS) were used to investigate the SEI chemistry. The disassembly of the coin cell and the preparation and characterization of the deposited Li on the Cu foil were all performed in an argon atmosphere to avoid a side reaction from the air. After discharging for 1 h at 1 mA cm^−2^, the surface components of the SEI films on the Cu electrodes using PP and GF consisted of a large amount of residual LiTFSI salt, few organic lithium alkyl carbonates and inorganic Li_2_CO_3_/Li_2_N*_x_*O*_y_*/Li_2_S*_x_*O*_y_* salts (detailed discussions in Supplementary Figs S17–S19) [53,54]. The difference from the case of using LiF–NFs-IL is that the signal of the inorganic LiF at ∼685 eV could be detected in the F1s spectrum (Fig. 5a) [42,55]. The cross-section SEM image demonstrates the tight contact between the deposited Li and the LiF–NFs-IL (Supplementary Fig. S20). Compared with the few S, N and O elements, the large amount of F element distribution on the deposited Li indicates that the signal of the inorganic LiF in the F1s spectrum is mainly originated from *insitu* coated LiF–NFs. To further investigate the inorganic inner layer near to the electrode/SEI interface, Ar^+^ depth-etching was performed. After etching for 60 s, the ratio of the inorganic LiF to the organic functional group of –CF_3_ at ∼688.8 eV dramatically increased. The C, O, N and S XPS spectra also revealed the existence of a few inorganic Li_2_O, Li_3_N and Li_2_S/Li_2_S_2_, and a decrease in organic components (Fig. 5b and Supplementary Fig. S21) [56–59]. When etched for 120 s, the contents of the inorganic salts in the SEI film did not change much, which indicates that the components of the inner SEI layer were uniform and stable. The SEI components of the deposited Li in the three cells after 10 cycles were further detected. It was found that the ratio of the inorganic LiF, Li_2_O and Li_3_N to the organic salts slightly increased when used a PP separator, owing to more decompositions of LiTFSI and DME/DOL solvents (Supplementary Fig. S22). In the case of using a GF separator, there was no significant change in the SEI components during the cycles (Supplementary Fig. S23). Interestingly, after 10 cycles, the LiF–NFs-IL induced a significant increase in inorganic LiF in the outer layer of the SEI film (Fig. 5c). When etched for 60 s and even 120 s, the main component in the inner layer was LiF–NFs, but inorganic Li_2_O, Li_3_N and the Li_2_S/Li_2_S_2_ had almost not been detected (Fig. 5d and Supplementary Fig. S24).

TOF–SIMS was used to further analyse the compositions and structures of the SEI layers of the Li-metal using a PP separator and LiF–NFs-IL after 10 cycles at 1 mA cm^−2^ with 1 mAh cm^−2^. The TOF–SIMS depth profiles and 3D rendering models showed the distribution of negatively charged fragments of the SEI on the Li-metal. When used a PP separator (Supplementary Fig. S25), organic CH^−^, C_2_H^−^, C_6_O_4_H_11_^−^ and LiNSO_2_F^−^ fragments could be principally detected on the surface of the SEI, which were attributed to the decomposition products of DME/DOL and LiTFSI salt. The signal of the C_2_F_6_S_2_O_4_N^−^ corresponded to the unreacted LiTFSI in the outer SEI layer. With the sputtering depth, the contents of the LiF_2_^−^, Li_2_F_3_^−^, LiO^−^ and SO_3_^2−^ fragments sharply increased, which were assigned to the inorganic LiF, Li_2_O and Li_2_SO_3_ phases. These inorganic products were formed by strongly electronegative atoms and metal lithium after the decomposition of the LiTFSI. This double-layer organic–inorganic structure of the SEI also agrees with the generally accepted model [60]. By contrast, the SEI structure in the case of using LiF–NFs-IL is different (Fig. 5e and f). The organic LiNSO_2_F^−^, C_2_F_6_S_2_O_4_N^−^ and C_6_O_4_H_11_^−^ fragments still form on the outer SEI film, but the thickness is larger than that in the general SEI film. Meanwhile, the LiF_2_^−^ and Li_2_F_3_^−^ contents were relatively high in the outer SEI film, which is attributed to the LiF–NFs. With the sputtering depth, the counts of organic CH^−^, C_2_H^−^and inorganic SO_3_^2−^ gradually increased, while the LiO^−^ count was always kept at low levels. These results are consistent with the XPS data. They demonstrate that the inner SEI film that used LiF–NFs-IL was an organic–inorganic composite phase consisting of large LiF–NFs and few inorganic and organic Li salts. The FIB-SEM image further verifies the cross-section structure that the ‘LiF–NFs-rich’ SEI film tightly covers on the surface of the deposited Li-metal (Supplementary Fig. S26). EIS analysis reveals that the formation of the ‘LiF–NFs-rich’ SEI leads to lower interfacial SEI resistance (*R_SEI_*) after the first discharge and charge at 1 mA cm^−2^ with 1 mAh cm^−2^ (Fig. 5g and h). In summary, the tortuous and discontinuous Li^+^ transfer interface between the organic layer and the uneven inorganic nanoparticles in a conventional SEI would lead to a sluggish Li^+^ migration (Fig. 5i). By contrast, the ‘LiF–NFs-rich’ SEI film could provide a continuous interface between the LiF–NFs and other organic/inorganic species, rendering a fast Li^+^ transport (Fig. 5j) [61]. Therefore, the voltage polarization of the Li//Li cell using LiF–NFs-IL could be largely decreased (Fig. 3c). The above results demonstrate that LiF–NFs-IL endows a Li-metal anode with a stable and high-conductivity SEI layer, which not only facilitates uniform Li plating/stripping, but also reduces the interfacial Li^+^ diffusion resistance.

The formation of a ‘LiF–NFs-rich’ SEI layer on Li-metal leads to the existence of a Li–LiF interface during Li deposition. In order to gain a deeper insight into the improvement mechanism of the Li–LiF interface for Li deposition, DFT calculation was employed to simulate the Li atom adsorption and diffusion behaviors in the interface between the LiF and the Li-metal. The mainly exposed (110) facet of the LiF and the (001) facet of the Li-metal were selected to build the thermodynamically stable configuration of the Li–LiF interface. Under the most stable Li atom adsorption configurations, the binding energy between the Li and Li–LiF interface could reach −2.14 eV (Fig. 5k), which is higher than the value of −1.52 eV for a bare Li (001) surface. It indicates that the Li–LiF interface can provide lithiophilic sites for reducing Li adsorption and nucleation energy barriers [62]. This result also explains the low *η*_n_ in the Li-metal batteries with LiF–NFs-IL (Fig. 3a). Bader analysis confirms that a Li atom can accept ∼0.22|e| from a metallic Li (001) surface, as shown in the charge density difference plots (Fig. 5l). By comparison, a Li atom donates ∼0.39|e| in a Li–LiF interface (Fig. 5m). This result indicates that the presence of a Li–LiF interface enhances the electron delocalization of the deposited Li atom, which promotes the formations of Li–Li metallic bonds and Li–F covalent bonds, thus inducing favorable Li adsorption and nucleation. The diffusion energy barrier of Li adatoms at the electrode surface plays a significant role in the uniformity of Li deposition. The nudged elastic-band method was used to investigate the Li diffusion behaviors on the Li (001) surface and Li–LiF interface. The Li diffusion path on the Li (001) surface along the *b*-axis is shown in Supplementary Fig. S27a, where the calculated energy barrier of the Li migration is 0.17 eV. To compare the energy barriers of the migrating Li across the Li–LiF interface and the Li (001) surface, the three Li diffusion paths along the *b*-axis, *a*-axis and [110] direction were used, which were named Path 1, Path 2 and Path 3, respectively (Fig. 5n and Supplementary Fig. S27b–d). The corresponding energy barriers are 0.22, 0.65 and 0.71 eV (Fig. 5o), respectively, which are larger than that of Li migration on a bare Li surface. The relationship between the activation energy and the rate of Li diffusion can be analysed using the Arrhenius equation [63]:

$$\begin{equation*}
k\, = \, A{e}^{ - {E}_a/RT},
\end{equation*}$$

where *k* is the rate constant, *A* is the frequency factor, *E_a_* is the activation energy, *R* is the gas constant and *T* is the absolute temperature (Kelvin). Under the same electrochemical environment, *k* is a function of *E_a_*. A higher value of *E_a_* leads to a lower growth rate of Li-metal, which facilitates uniform Li deposition. In the case of the Li atom on the Li (001) surface, low binding energy can induce an enhanced propensity to form clusters, while the low activation energy barrier increases the tendency for migration of adatoms from adsorption sites to regions of faster growth. By comparison, in the Li–LiF interface, the higher binding energy and activation energy favor a more uniform Li nucleation and a lower tendency for the over-growth of Li dendrites.

**Figure 6.**
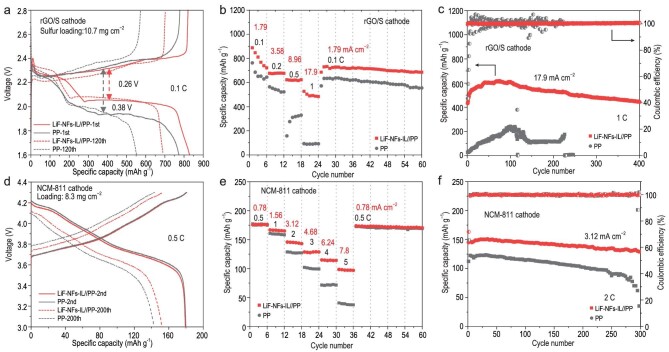
Electrochemical performance of lithium-metal batteries. The electrochemical performances of high-loading Li//rGO–S coin cells with and without LiF–NFs-IL: (a) charge–discharge curves at the 1st and 120th cycles at 0.1 C, (b) rate performance and (c) cycling stability at 1 C. The high-loading Li//NCM-811 coin cells: (d) charge–discharge curves at the 2nd and 200th cycles at 0.5 C, (e) rate performance and (f) cycling stability at 2 C.

To demonstrate the application potential of LiF–NFs-IL in practical LMBs, the electrochemical performances of high-loading Li//rGO–S and Li//LiNi_0.8_Co_0.1_Mn_0.1_O_2_ (NCM-811) cells were investigated. During the assembly process of the LMBs, LiF–NFs-IL was put between the PP separator and the Li-metal anode. For the Li–S battery, the rGO xerogel/S cathode with high sulfur content (70 wt%) and high sulfur loading (10.7 mg cm^−2^) was prepared by facile melting of sulfur at 155^o^C for 12 h. In the as-synthesized cathode, the S nanoparticles are uniformly distributed in the rGO xerogel (Supplementary Fig. S28). At 0.1 C, LiF–NFs-IL renders a higher discharge specific capacity of 828.5 mAh g^−1^ (8.86 mAh cm^−2^) and a lower polarization voltage of 0.26 V, as compared with those of the Li//rGO–S cell only using a PP separator (773.6 mAh g^−1^, 0.38 V) (Fig. 6a). That is ascribed to the reduced *R_ct_* and *R_SEI_* after the formation of the Li–LiF interface. After 120 cycles, the Li//LiF–NFs-IL//PP//rGO–S cell still retains a specific capacity of 694.3 mAh g^−1^ and the retention rate is 83.8% (Supplementary Fig. S29). However, the capacity retention rate of the rGO–S//PP//Li cell is only 71.4%. The rate performances of the two cells were further tested and it was found that the specific capacity of the Li//PP//rGO–S cell dropped sharply at 1 C (ultra-high area current density of 17.9 mA cm^−2^) and only 98 mAh g^−1^ could be delivered (Fig. 6b). This is due to the high area current density leading to serious voltage polarization and capacity loss in the case of using a PP, which is consistent with the result of the symmetrical Li//PP//Li cell (Fig. 3c). Excitingly, the Li//LiF–NFs-IL//PP//rGO–S cell can still display a high specific capacity of 528 mAh g^−1^ at 1 C. The high area capacity of 5.65 mAh cm^−2^ at 17.9 mA cm^−2^ has largely exceeded the level of commercial Li-ion cells (3–4 mAh cm^−2^) [64–66] and the most recent work (Supplementary Table S1). After 400 cycles, the Li//LiF–NFs-IL//PP//rGO–S cell still maintains a specific capacity of 444.6 mAh g^−1^, which is 72% of the highest specific capacity during cycling, while the CE can be kept at 100% (Fig. 6c). However, the capacity of the Li//PP//rGO–S cell rapidly fades after 120 cycles and it suffers from short circuit after 225 cycles.

When paired with a high-loading NCM-811 cathode (8.3 mg cm^−2^), the LiF–NFs-IL coating the Li-metal anode endows a high capacity retention of 85% and low voltage hysteresis at 0.5 C (1 C = 188 mA g^−1^) after 200 cycles (Fig. 6d and Supplementary Fig. S30). Especially for rate performance, the Li//LiF–NFs-IL//PP//NCM-811 cell exhibits largely enhanced capacities of 145.9, 128.9, 115 and 98.6 mAh g^−1^ at 2, 3, 4 and 5 C, respectively (Fig. 6e). It was also verified that LiF–NFs-IL could reduce voltage polarization and Li^+^ diffusion resistance at a high area current density. At 2 C, the Li//LiF–NFs-IL//PP//NCM-811 cell also delivers better cycling stability for 300 cycles compared with that of the Li//PP//NCM-811 cell (Fig. 6f). To further demonstrate the potential of LiF–NFs-IL for commercial application, the ‘anode-free’ Cu//LiF–NFs-IL//PP//NCM-811 pouch cell with three layers (cathode) was packaged (Supplementary Fig. S31). It displays an area capacity of 1.2 mAh cm^−2^ in the first discharge. The initial CE of the Cu//LiF–NFs-IL//PP//NCM-811 pouch cell can reach 80%, which is close to that of a Li//LiF–NFs-IL//PP//NCM-811 coin cell (Supplementary Fig. S32), contributed by the uniform and high-efficiency deposition/dissolution behaviors of Li on Cu coated by LiF–NFs-IL. The second CE of 96.9% is consistent with that of the Li//LiF–NFs-IL//Cu coin cell. Meanwhile, the reversible cycles of the ‘anode-free’ pouch cell suggest the large potential of LiF–NFs-IL for practical LMBs.

## CONCLUSIONS

In summary, millimeter-level and single-crystal LiF nanofibers with uniquely serrated morphologies were easily prepared using freeze-drying. The ‘ice-sublimation’-induced vertical crystallization growth mechanism of LiF nanofibers along the <111> orientation was demonstrated by electron microscopy characterizations. The porous LiF–NFs interlayer exhibits excellent thermal stability, wettability and a high Li^+^ transference number. When used in a Li-metal battery, it induces the formation of a ‘LiF–NFs-rich’ SEI. The continuous Li^+^ transport interface between the LiF–NFs and the organic/inorganic species decreases the SEI resistance. Another Li–LiF interface between the SEI and the Li-metal provides lithiophilic sites for reducing the Li^+^ adsorption and nucleation energy barriers. The high diffusion energy barriers of Li^+^ between the Li–LiF interface lead to a low Li growth rate, facilitating flat and dense Li deposition. Thus, a low overpotential (11 mV), high current density (12 mAh cm^−2^) and stable Li plating/stripping for >1600 h under 4 mAh cm^−2^ can be achieved. When paired with high-loading rGO–S and NCM-811 cathodes, the LiF–NFs interlayer enables significantly improved cycling life and specific capacities at high rates compared with the bare Li anode. The LiF–NFs interlayer can be easily integrated into various liquid and even solid LMBs, exhibiting great potential for commercial application.

## METHODS

### Material synthesis

#### Preparation of LiF nanofibers

First, 503.56 mg (12 mmol) of lithium hydroxide monohydrate powder was dissolved in 200 mL of deionized water and stirred at room temperature for 15 min. After the completed dissolution of the above solution, 600 μL of hydrofluoric acid (≥40 wt%) was slowly added and stirring was continued at room temperature for 15 min. Then the above transparent solution was poured into a plastic surface dish with a diameter of 14 cm, which was placed in a refrigerator at −40^o^C for freezing for 24 h. The frozen sample was quickly transferred to a freeze dryer and dried for 24 h with a vacuum degree of 5–10 Pa. Then, a ‘cotton-like’ LiF xerogel was be obtained, composed of ultra-long LiF nanofibers. The LiF cubic particles were prepared using the common drying of LiF transparent aqueous solution at 70^o^C.

#### Preparation of LiF nanofibers interlayer

First, 50 mg of LiF nanofibers were dispersed in 20 mL of alcohol and stirred for 20 min at room temperature to form a uniform mixed solution. Then, the mixed solution was placed on a GF membrane with a diameter of 4 cm for vacuum filtration to obtain the free-standing LiF–NFs-IL. The thickness of the LiF–NFs-IL could be controlled by adjusting the mass of LiF nanofibers in the mixed solution.

### Materials characterization

XRD data were collected with a D2 Advance X-ray diffractometer using Cu Kα radiation (λ = 1.5418 Å). The microstructures were observed using FESEM (JEOL-7100F). TEM, HRTEM images and SAED patterns were recorded by using a Talos F200S microscope operated at 200 kV. For XPS, the samples were prepared and transferred to the chamber of an ultra-high vacuum Imaging XPS Microprobe system for analysis (Kratos Axis SUPRA) in an argon-filled glove box. The in-depth spectra from top to bottom were collected after Ar sputtering; the putter etching was performed using an Ar^+^ beam (4 keV and 140 μA) and the sputtering rate was ∼10 nm/min on Ta_2_O_5_. All spectra were fitted with Gaussian–Lorentzian functions and a Shirley-type background using XPSPEAK software. The *in situ* optical visualization observations were conducted on an optical microscope equipped with electron multiplying CCD and monochromator. TG curves were performed using a STA449F3 integrated thermal analysis instrument (Netzsch, Germany) under an air environment at a rate of 10°C min^−1^. TOF–SIMS measurements were conducted using a PHI nano TOF II. A Bi_3_^++^ beam (30 keV, 2 nA, 5 × 5 μm^2^) was used as the primary beam to detect the samples; sputtering with an Ar^+^ beam (3 keV, 100 nA, 400 × 400 μm^2^) was applied for depth profiling analysis. The sputtering rate was ∼0.17 nm/s on SiO_2_.

### Electrochemical measurements

Galvanostatic charge/discharge measurements were performed using multichannel battery testing systems (LAND CT2001A and NEWARE CT-4000Tn). EIS was measured in the frequency range of 0.01 Hz–100 kHz with an AC perturbation signal of 10 mV. EIS and CA tests were all conducted using an EC-lab electrochemical workstation. All measurements were carried out at room temperature.

## Supplementary Material

nwac183_Supplemental_Files
