# Supplementary material for: Serrated lithium fluoride nanofibers-woven interlayer enables uniform lithium deposition for lithium-metal batteries
Source: Natl Sci Rev. 2022 Sep 1;9(11):nwac183. doi: 10.1093/nsr/nwac183 (PMC9647010; doi:10.1093/nsr/nwac183)
Supplement: nwac183_Supplemental_Files [file nwac183_supplemental_files.zip › Supplementary_data.docx]

Supporting Information

Serrated lithium fluoride nanofibers-woven interlayer enables uniform lithium deposition for lithium metal batteries

Shuangshuang Tan,^1,2^ Yalong Jiang,^1^ Shuyan Ni,^1^ Hao Wang,^1^ Fangyu Xiong,^1^ Lianmeng Cui,^1^ Xuelei Pan,^1^ Chen Tang,^1^ Yaoguang Rong,^4^ Qinyou An^1,3,*^ and Liqiang Mai^1,3,*^

^1^State Key Laboratory of Advanced Technology for Materials Synthesis and Processing, Wuhan University of Technology, Wuhan 430070, China;

^2^College of Materials Science and Engineering, Chongqing University, Chongqing 400030, China;

^3^Foshan Xianhu Laboratory of the Advanced Energy Science and Technology Guangdong Laboratory, Foshan 528200, China;

^4^ Wuhan National Laboratory for Optoelectronics, Huazhong University of Science and Technology Wuhan 430074, China

***Corresponding authors**. anqinyou86@whut.edu.cn; mlq518@whut.edu.cn

**METHODS**

**Material synthesis**

*Preparation of rGO-S cathode:* 500 μL of NH_3_·H_2_O was added into 5 mL graphene oxide aqueous solution (2 mg mL^−1^). After 10 min stirring, the mixed solution was transferred to a 10 mL Telfon-lined autoclave and hydrothermally treated at 180^o^C for 12 h. Finally, the obtained GO hydrogel sample was freeze-dried and calcinated at 550 °C for 2 h under a flowing ammonia atmosphere. The as-prepared rGO xerogel with a diameter of 0.6 cm was uniformly sheared to a self-supporting xerogel electrode about 1.3 mg per portion. Then 31 µL of S/CS_2_ solution (0.1 mg µL^−1^) was dropped into the xerogel electrode. After CS_2_ was completely volatilized, the xerogel electrode was placed into a 10 mL Telfon-lined autoclave and heated at 155^o^C for 12 h. After cooling to room temperature, the rGO-S (sulfur content of 70 wt%) cathode could be obtained. The sulfur loading was calculated to be approximately 10.7 mg cm^−2^.

**Electrochemical measurements**

For Li//Cu cells, the electrochemical properties were characterized in 2025-type coin cells with Li metal foil as the anode and Cu foil (a diameter of 16 cm) as the cathode. The types of PP and GF separators are Celgard 2400 and GF/A, respectively. The electrolyte was 1 M LiTFSI dissolved in DOL and DME (1:1 ratio by volume) with 0.2 M LiNO_3_ as the additive. The assemblings of Li//Li cells and high-loading Li//rGO-S cells were consistent with Li//Cu cells, except for the cathode. For Li//NCM-811 cells, the NCM-811 cathode with active material of 94.5 wt% and area loading of 8.3 mg cm^−2^ was purchased from Guangdong Canrd New Energy Technology Co.,Ltd. The electrolyte was 1 M LiPF_6_ in 1:1 v/v EC/DEC with 5 wt% FEC. LiF-NFs-IL with a diameter of 17 cm was placed between the separator and lithium metal anode. The thickness of lithium foil is 0.4 mm. The actual amounts of electrolytes in all coin cells were ~5 μL mg_actived cathode_^−1^. All cells were assembled in an argon-filled glove box.

**DFT calculations**

All calculations were performed by using the projector augmented wave (PAW) method within the DFT as implemented in the Vienna ab initio simulation package (VASP). The generalized gradient approximation (GGA) in the forms of Perdew-Burke-Ernzerhof (PBE) was used to treat the exchange-correlation energy. DFT-D3 scheme was applied to account for the van der Waals interactions during the calculations, and the plane wave basic sets cut off energy is 500 eV. A slab model of 2×2×1 supercell with three atomic layers was constructed to simulate the (001) facet of Li, then the binding energy between Li adatom and substrates (Li001) could be calculated. In order to demonstrate the effect of LiF on lithium deposition, a slab model of a 2×2×1 supercell with three atomic layers, simulating the (110) facet of LiF, was added to the Li001 slab (Li001/LiF110 with a distance of about 5.3 Å). A vacuum slab of about 10 Å was inserted between the surface slabs for all the models. For the Brillouin-zone sampling, 2×2×1 of k-point was set for the structure relaxation and increased to 4×4×1 for the electronic structure calculations. Except for the bottom two atomic layers of the Li001, all atoms were allowed to be fully relaxed while keeping the supercell boxes unchanged until the residual force per atom was less than 0.05 eV Å^‒1^. While for Li001/LiF110, the bottom two atomic layers of the Li001 and top two atomic layers of the LiF110 were fixed, and other atoms were allowed to be fully relaxed while keeping the supercell boxes unchanged until the residual force per atom was less than 0.05 eV Å^‒1^. Ultrasoft pseudopotentials were used to describe the interaction of ionic core and valence electrons. The binding energies (E_b_) between Li and substrates (Li001 and Li001/LiF110) are defined by equation:

E_b_= E_Total_−E_Substrate_-E_Li_

Where E_Total_, E_Substrate_ and E_Li_ are the total energies of a composite system of the substrate with Li, substrate and an isolated Li, respectively. Site 1 (under Li of LiF), site 2 (under F of LiF) and site3 (lies the halfway between Li and F of LiF) were chosen to simulate the optimal adsorption sites. More negative values of the binding energy indicate that the Li prefers to be adsorbed. Then, the Li migration pathway on substrate and energy barrier were optimized with nudged elastic band (NEB) method. Path 1 (along the *b* axis), Path 2 (along the *a* axis), Path 3 (along the diagonal ([110] direction)) were considered.


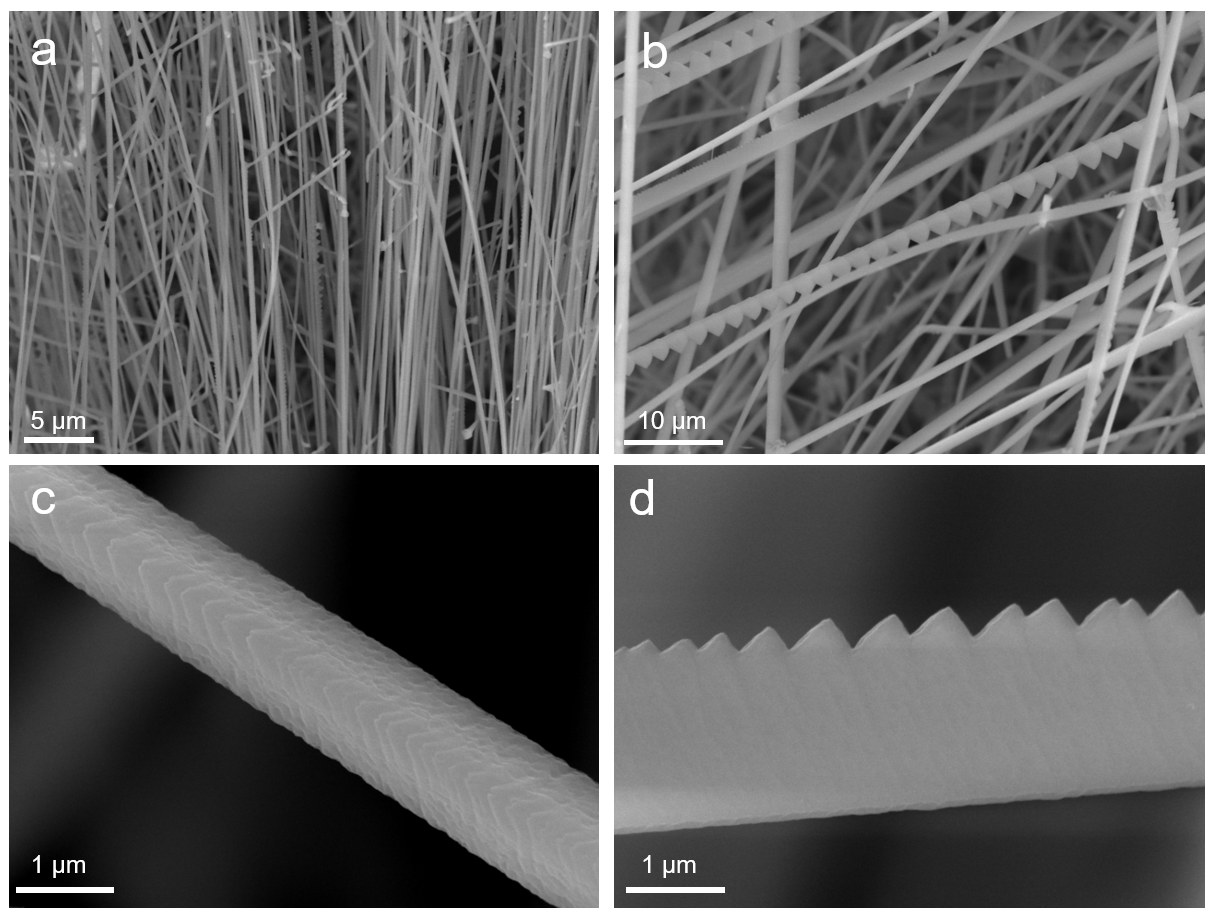


**Figure S1**. SEM images of serrated LiF-NFs.


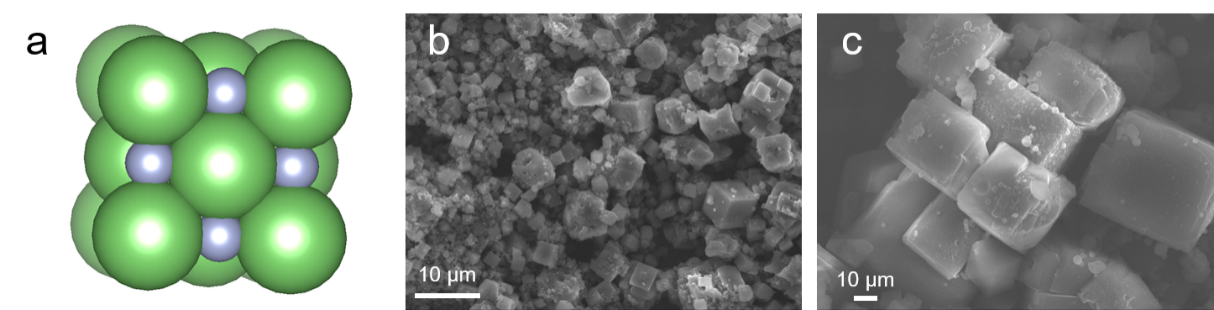


**Figure S2**. (a) Crystal structure of LiF. (b, c) SEM images of cubic LiF particles prepared by routine drying.

**
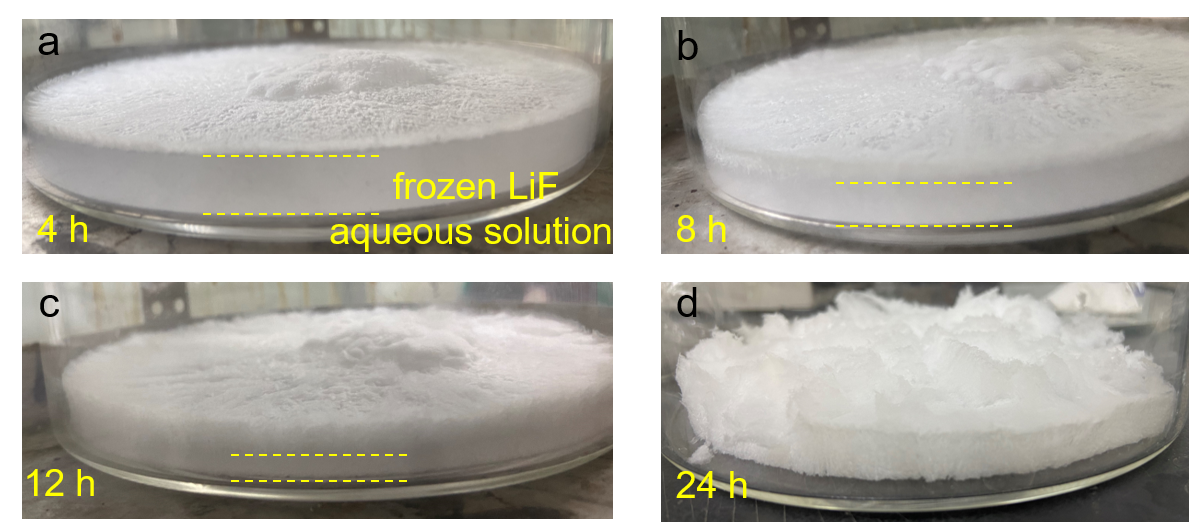
**

**Figure S3**. The optical images of the frozen LiF aqueous solution at different times during the freeze-drying process: 4 h (a), 8 h (b), 12 h (c) and 24 h (d).


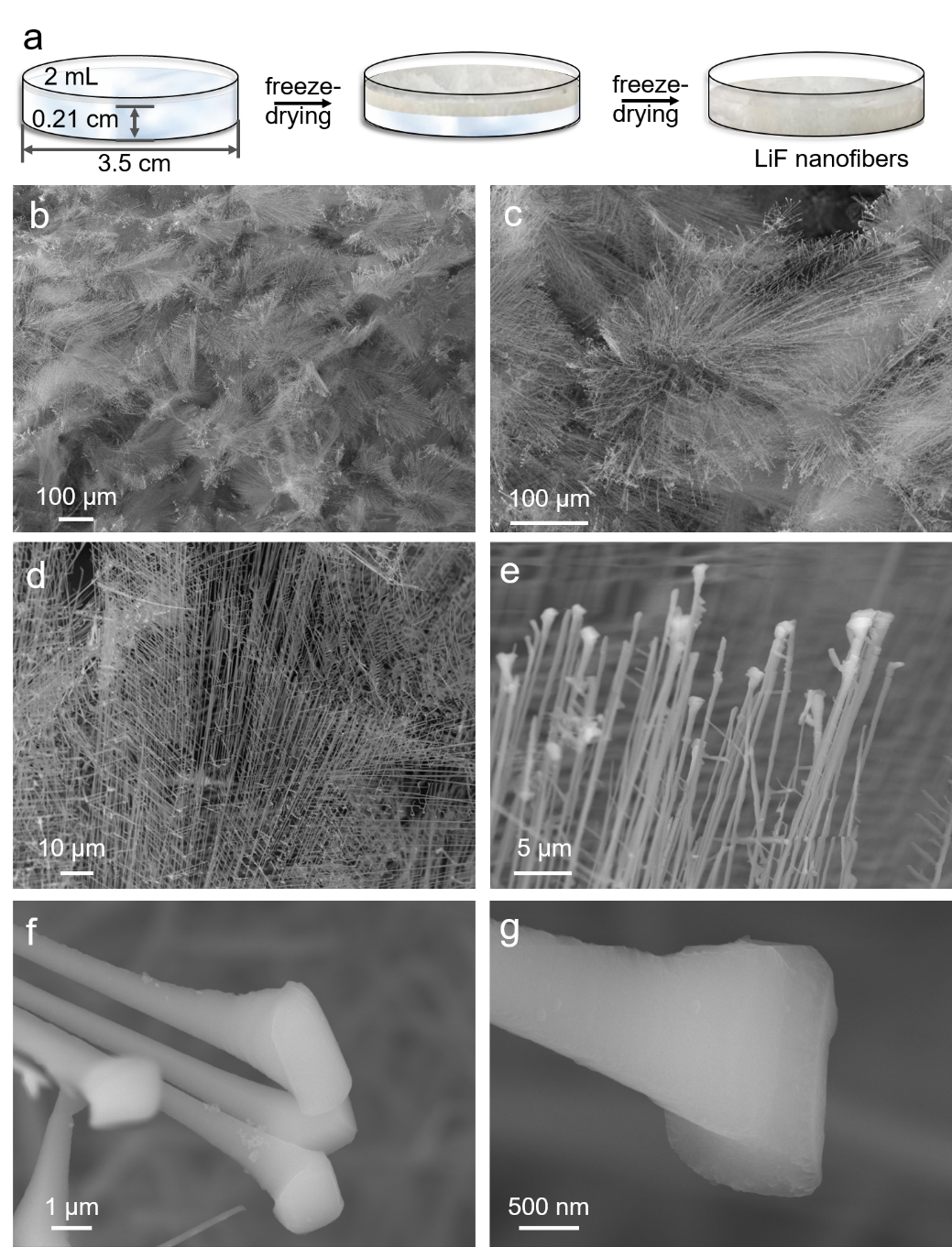


**Figure S4**. (a) The freeze-drying process of a thin LiF-NFs film with a thickness of approximately 2 mm. (b-e) the top SEM images of the thin LiF-NFs film. (f, g) SEM images of the end of LiF-NFs.

**
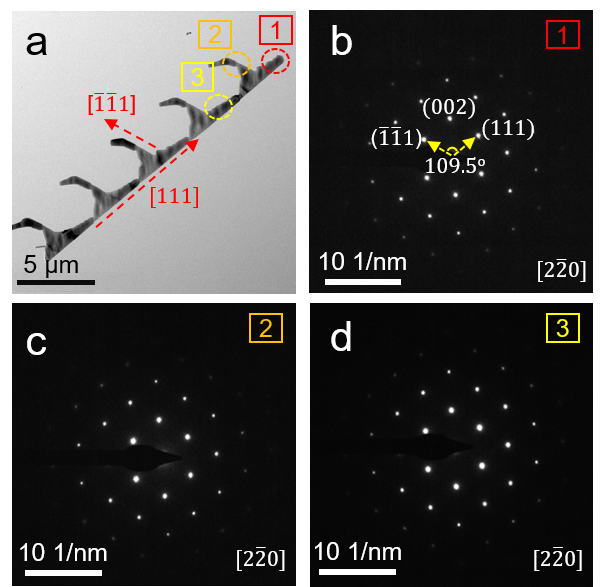
**

**Figure S5**. (a) TEM image of LiF-NF with branches. (b-d) The corrsponding SAED patterns of LiF-NF‘s trunk and branch.

**
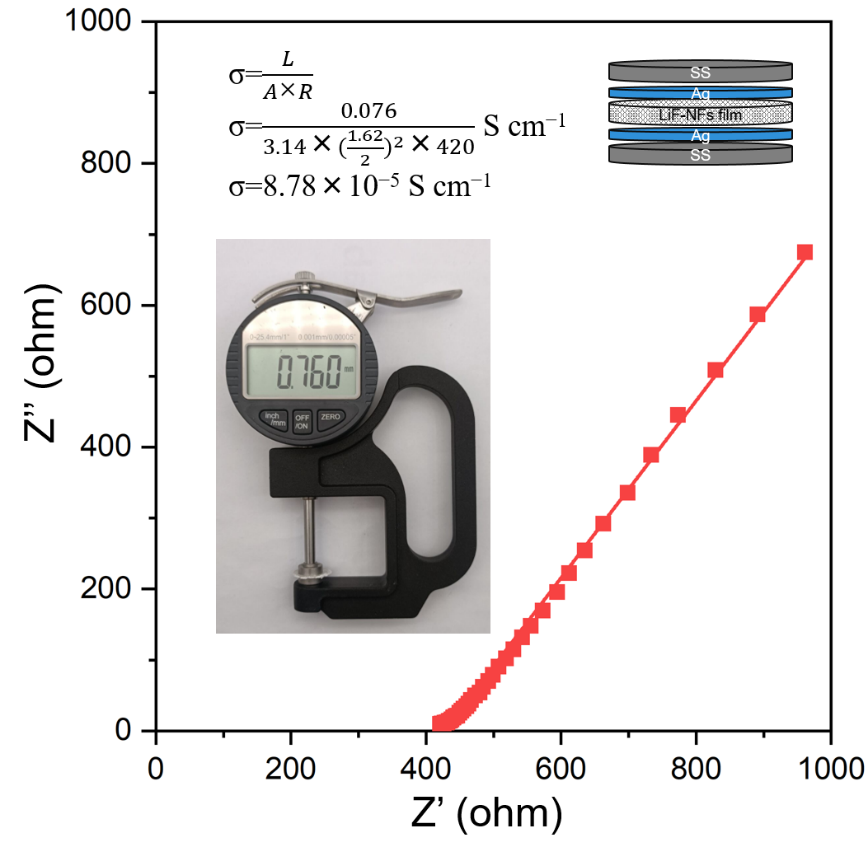
**

**Figure S6.** EIS profile of the LiF-NFs film compressed by 5 MPa at 25^o^C, inset is the thickness measurement of the LiF-NFs film.

The ionic conductivity of LiF-NFs film could be calculated according to the following equation: σ=L/(A·R), where σ is the ionic conductivity, A is the area of the ionic conductor, R is the resistance and L is the length of the ionic conductor. The thickness and diameter of LiF-NFs film are 0.076 and 1.62 cm, respectively.

σ=$\frac{0.076}{3.14\text{×}{(\frac{1.62}{2})}^{2}\text{×}420}$ S cm^−1^

σ=8.78×10^−5^ S cm^−1^


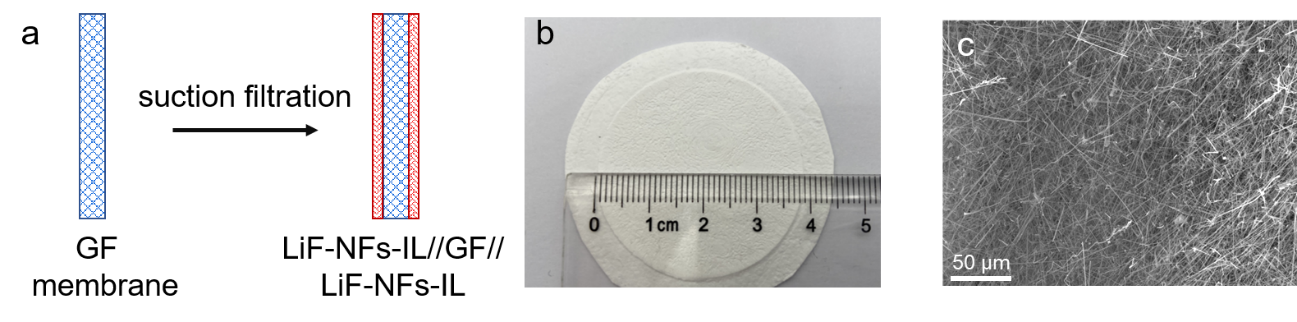


**Figure S7**. (a) Schematic of the preparation of LiF-NFs-IL by suction filtration. (b) Optical photo and (c) SEM image of LiF-NFs-IL.


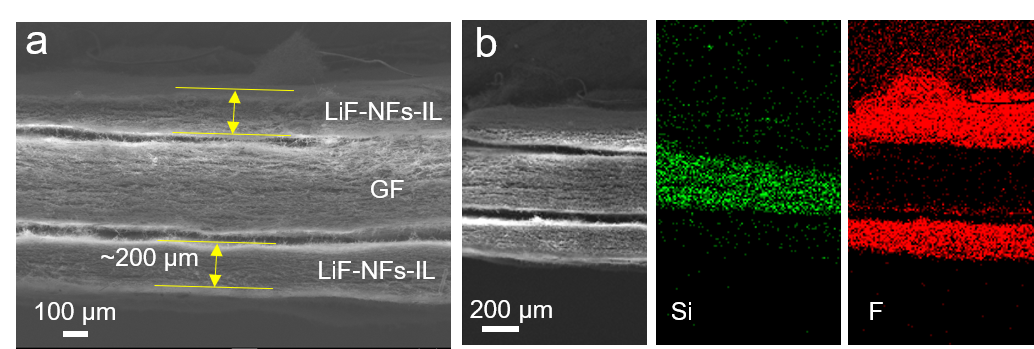


**Figure S8**. (a) SEM images and (b) EDS mapping of the cross-section of LiF-NFs-IL.


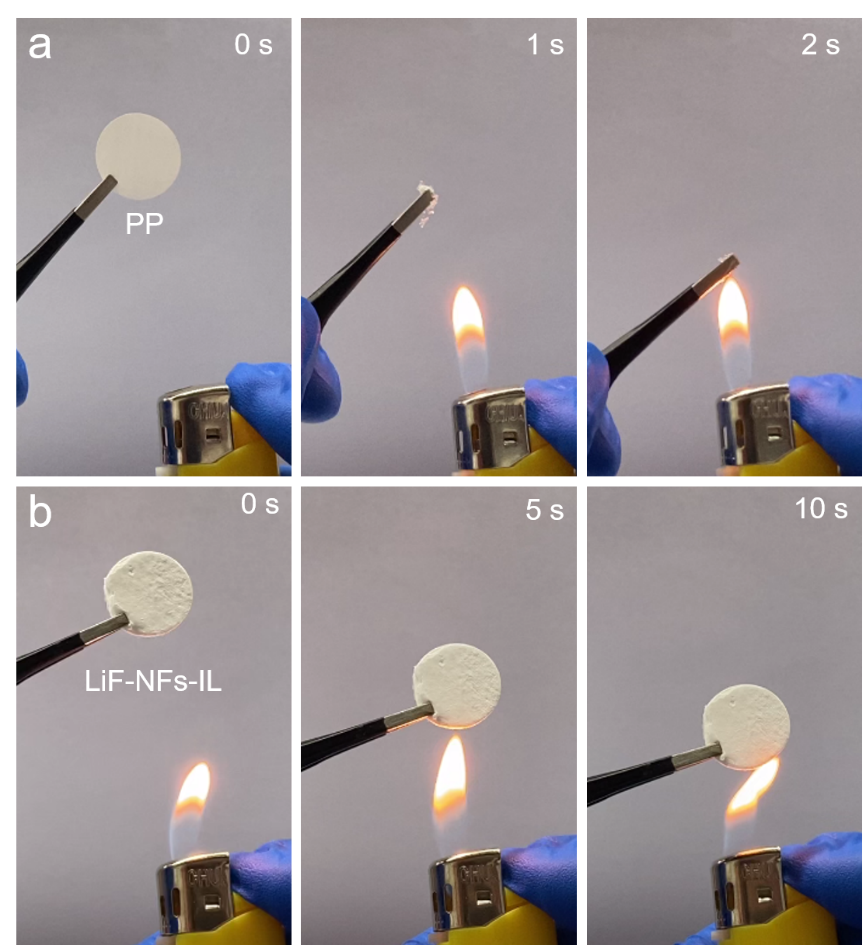


**Figure S9**. The burning tests for traditional PP separator (a) and LiF-NFs-IL (b).


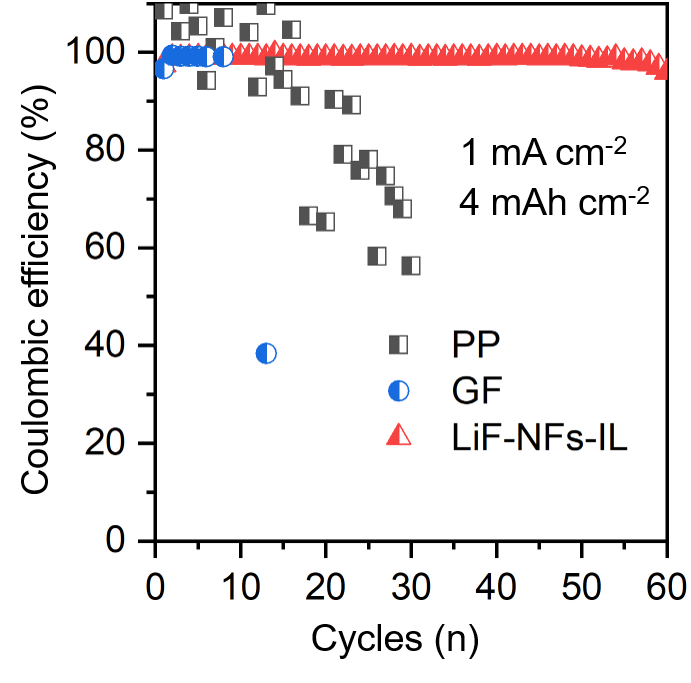


**Figure S10**. Cycling performances of Li//Cu cells with PP, GF and LiF-NFs-IL at 1 mA cm^−2^ with 4 mAh cm^−2^.


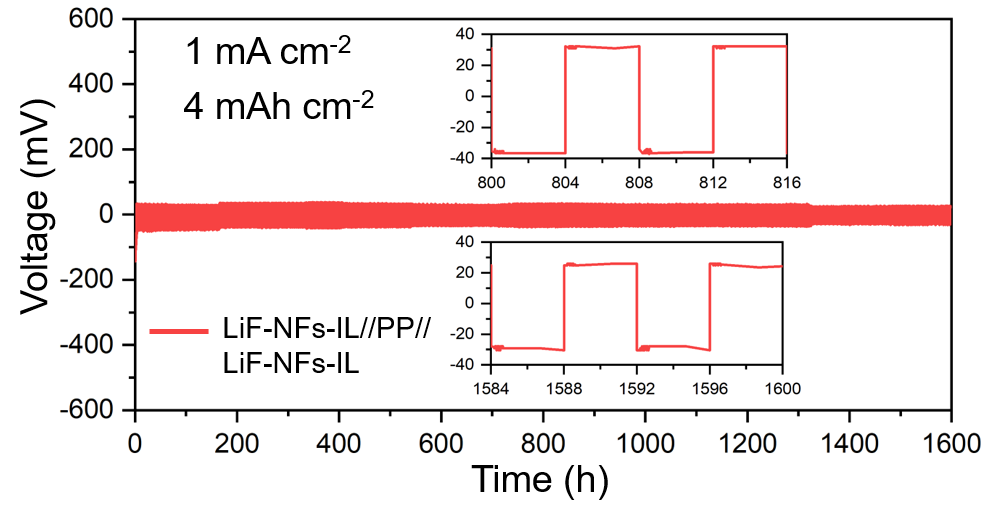


**Figure S11**. Cycling stabilities of Li//LiF-NFs-IL//PP//LiF-NFs-IL//Li cells at 1 mA cm^−2^ with 4 mAh cm^−2^.


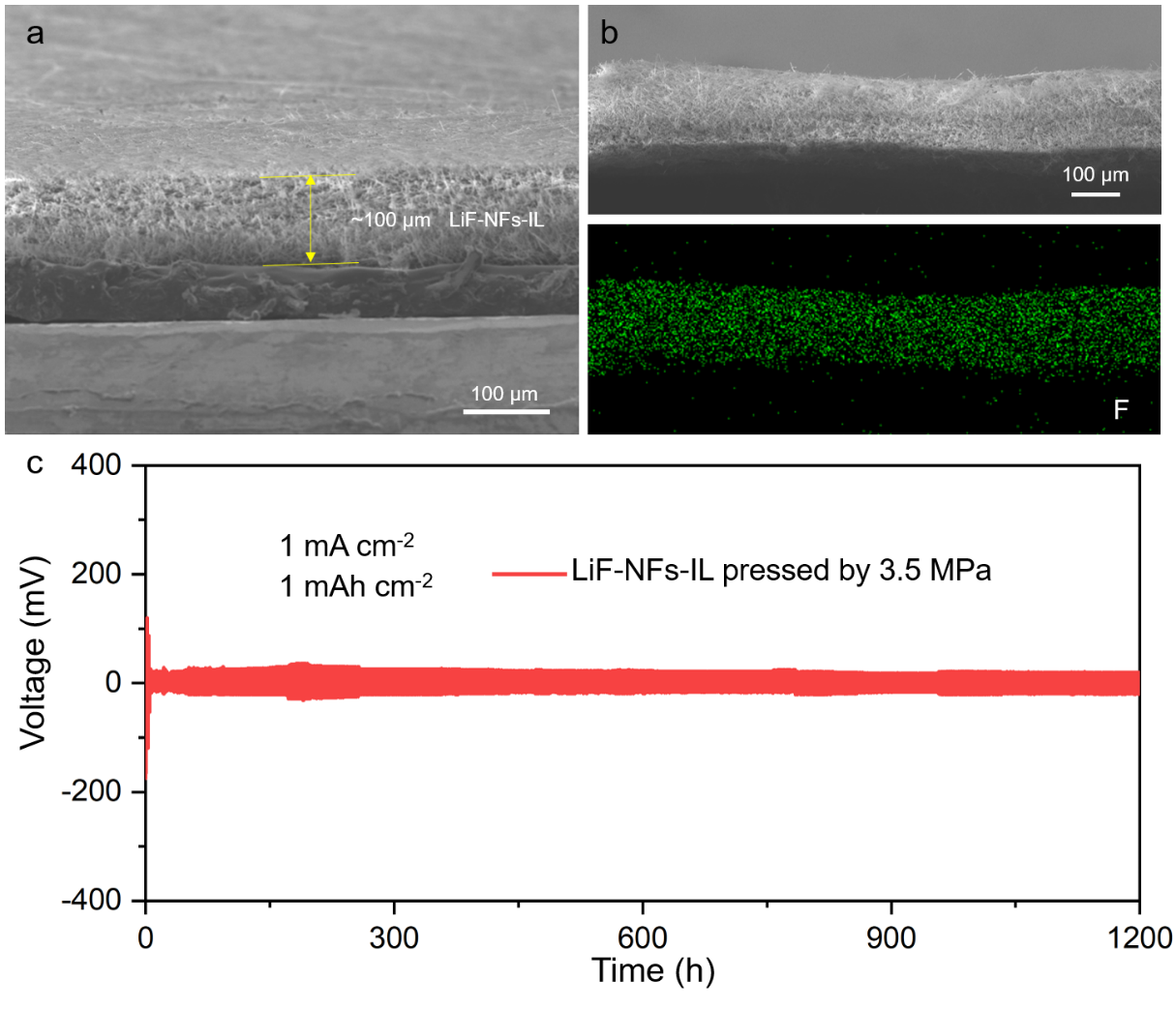


**Figure S12**. SEM image (a) and EDS mapping of the cross-section of the pressed LiF-NFs-IL by a pressure of 3.5 MPa. (c) Cycling stabilities of Li//Li cells with the pressed LiF-NFs-IL at 1 mA cm^−2^ with 1 mAh cm^−2^.


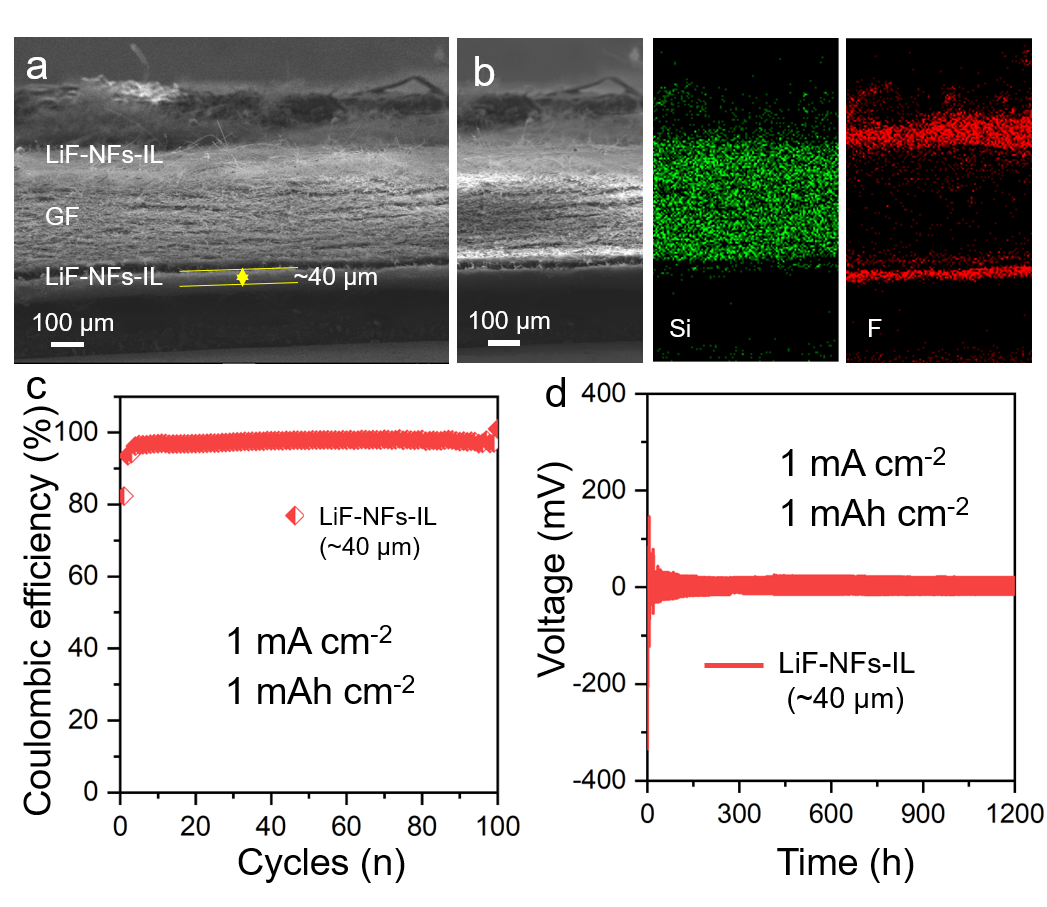


**Figure S13.** SEM image (a) and EDS mapping of the cross-section of the LiF-NFs-IL with a thickness of ~40 μm. (c) CE and cycling stabilities of Cu//Li cells. (d) Cycling stabilities of Li//Li cells at 1 mA cm^−2^ with 1 mAh cm^−2^.


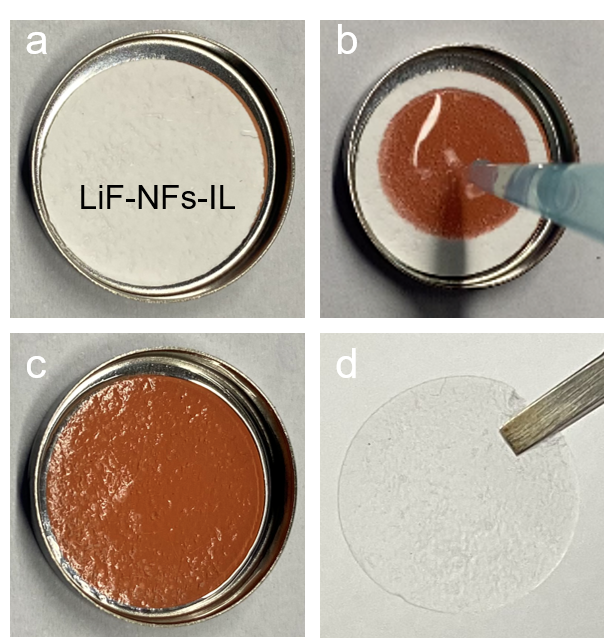


**Figure S14**. (a-d) Optical images of the LiF-NFs-IL coated on Cu foil during the electrolyte infiltration process.

**
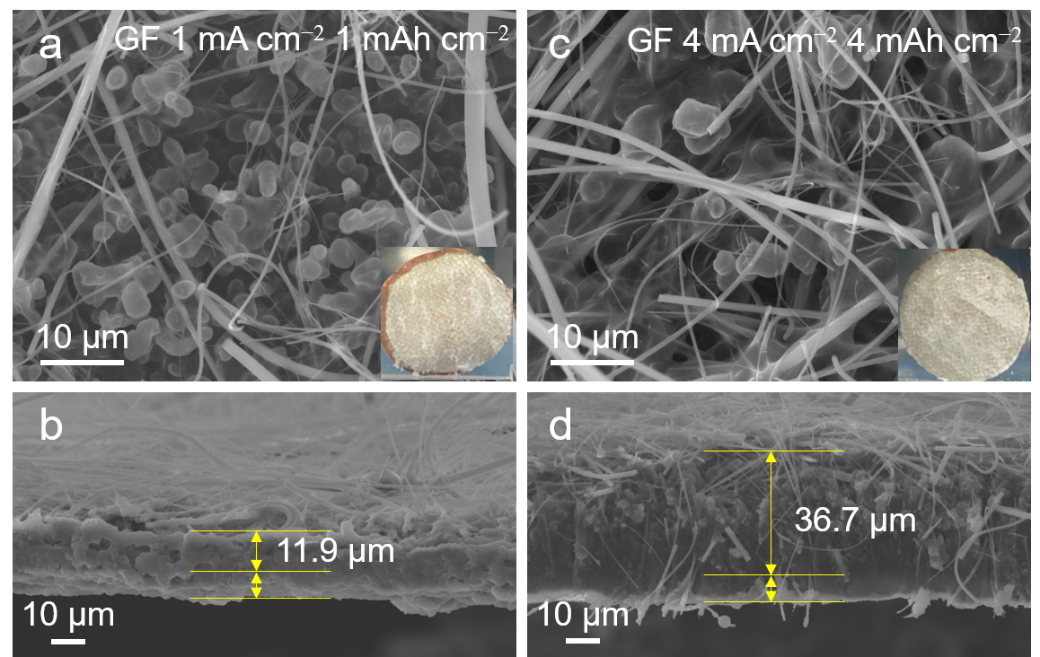
**

**Figure S15**. The frontal and cross-section SEM images of Li deposition on Cu foil at 1 mA cm^−2^ with 1 mAh cm^−2^ (a, b) and 4 mA cm^−2^ with 4 mAh cm^−2^ (c, d) using GF separator.

**
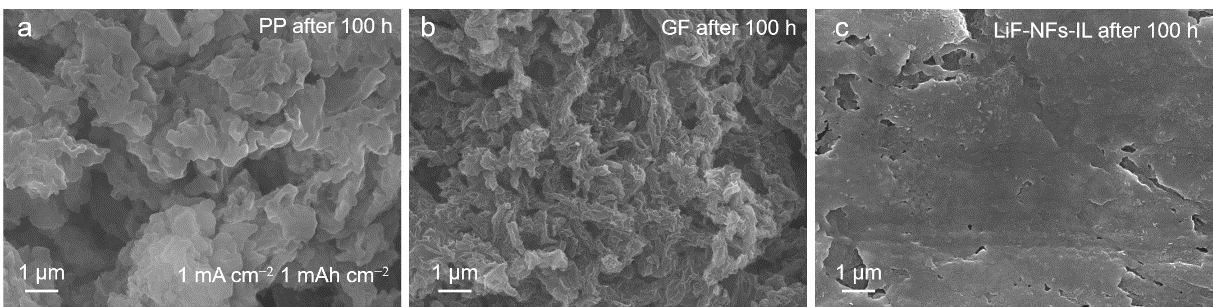
**

**Figure S16**. The surface morphologies of Li metal anodes in symmetrical Li//Li cells with PP (a), GF separators (b) and LiF-NFs-IL (c) at 1 mA cm^−2^ with 1 mAh cm^−2^ after 100 h.

**
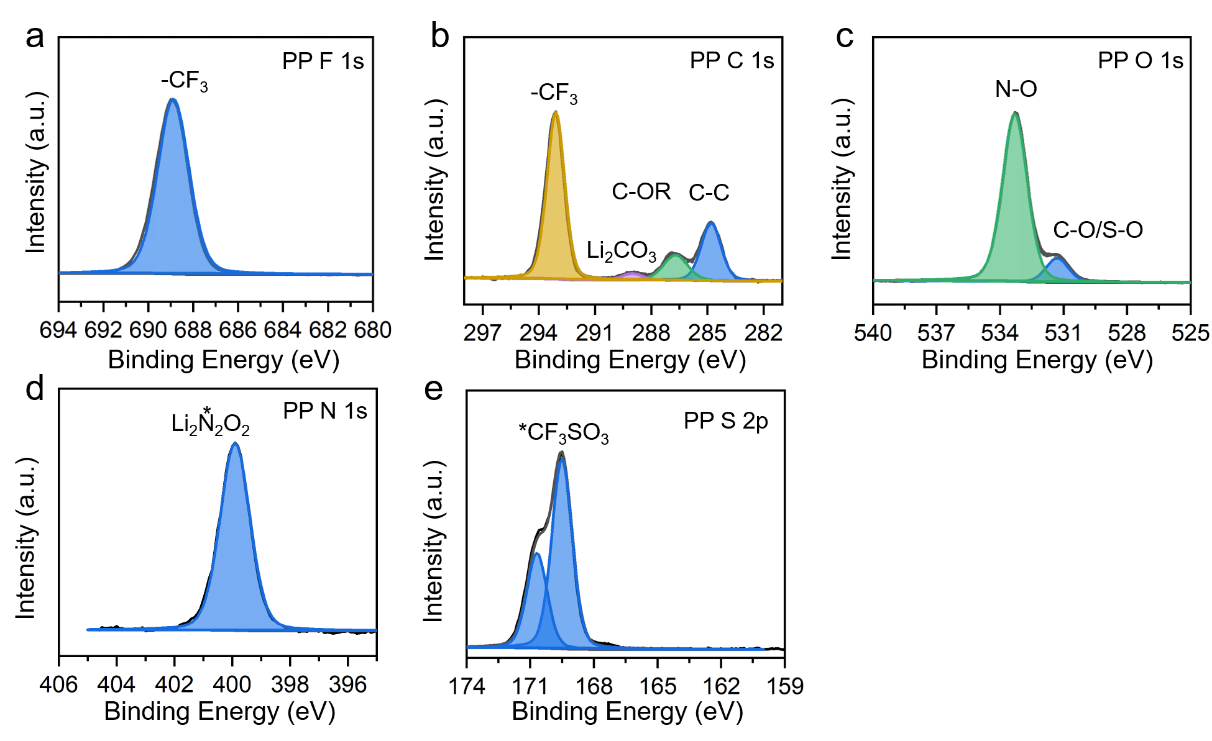
**

**Figure S17**. XPS F 1s (a), C 1s (b), O 1s (c), N1s (d) and S 2p (e) spectra of the deposited Li metal on Cu foil in Li//PP//Cu cell after discharging 1 h at 1 mA cm^−2^.

The peak at 688.8 eV in F 1s spectrum is ascribed to the -CF_3_ group of LiTFSI salt. In C 1s spectrum, the peaks at 293.1, 289, 286.7 and 284.8 eV are assigned to -CF_3_, Li_2_CO_3_, organic lithium alkyl carbonates and C-C bond, respectively. The peaks at 533.3 and 531.3 eV are attributable to the N-O and C-O/S-O bonds in inorganic Li_2_CO_3_/Li_2_N*_x_*O*_y_*/Li_2_S*_x_*O*_y_* salts. In N 1s spectrum, the peak at 399.9 eV is assigned to Li_2_N_2_O_2_ salt. In S 2p spectrum, the peak at 169.5 eV is the 2p 3/2 orbit of LiTFSI.

**
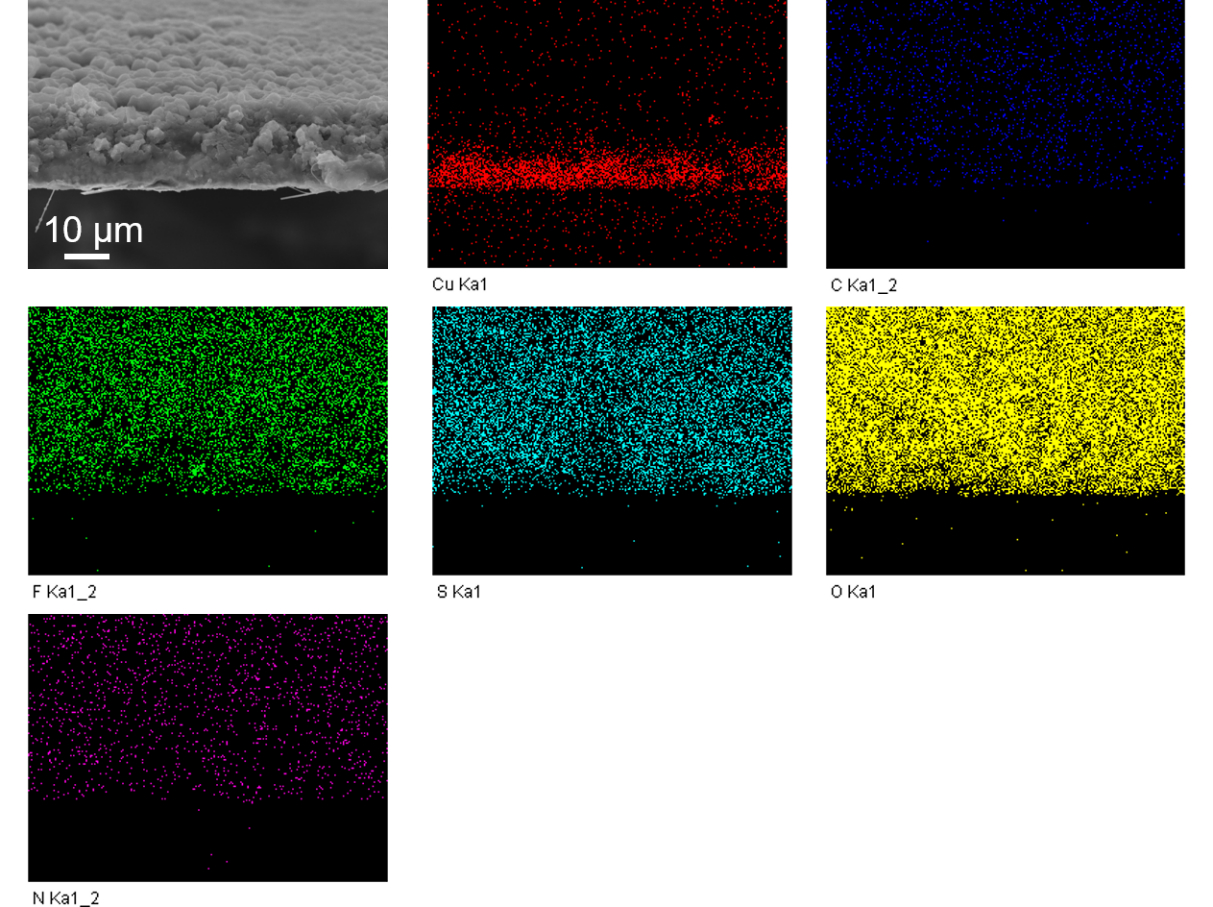
**

**Figure S18**. The cross-section SEM image and EDS mapping of the deposited Li metal on Cu foil in Li//PP//Cu cell after discharging 1 h at 1 mA cm^−2^.

Combined with XPS results, the uniform distribution of F, S, O and N elements may be contributed to a large number of residual LiTFSI salt and less decomposition products.

**
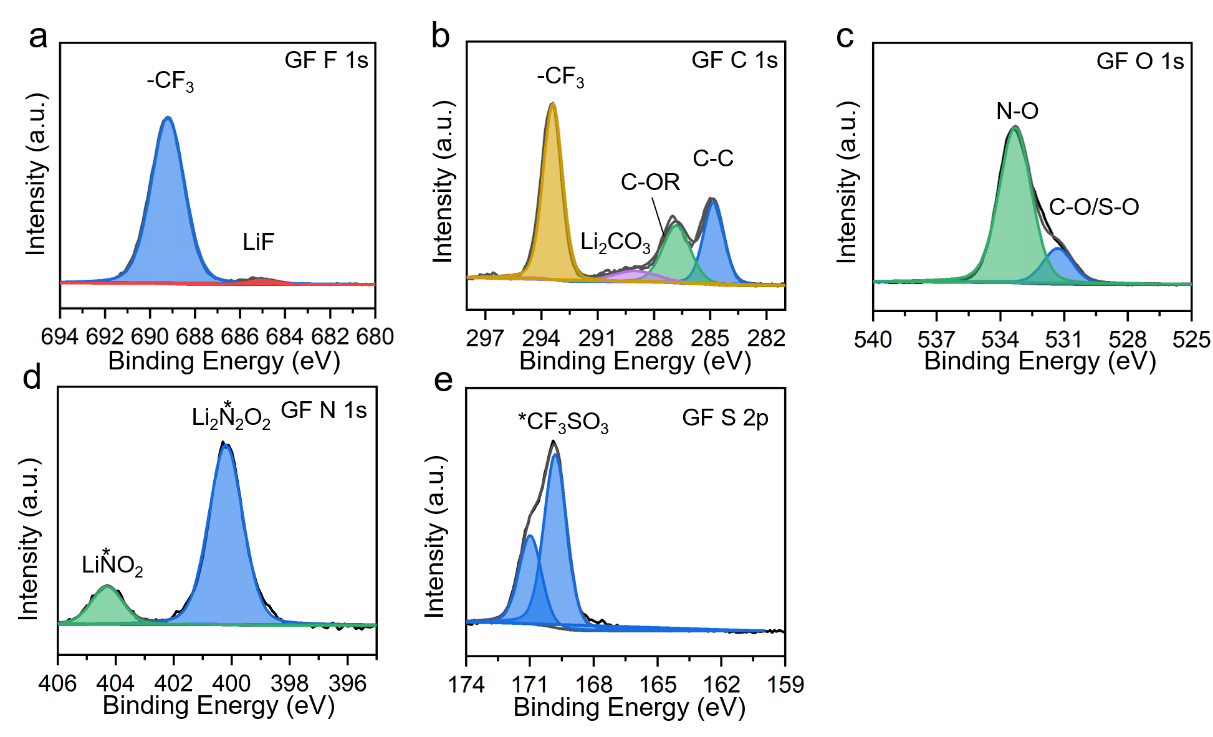
**

**Figure S19**. XPS F 1s (a), C 1s (b), O 1s (c), N1s (d) and S 2p (e) spectra of the deposited Li metal on Cu foil in Li//GF//Cu cell after discharging 1 h at 1 mA cm^−2^. The peak at 685 eV is ascribed to inorganic LiF. The peak at 404.3 eV is assigned to Li_2_NO_2_ salt.

**
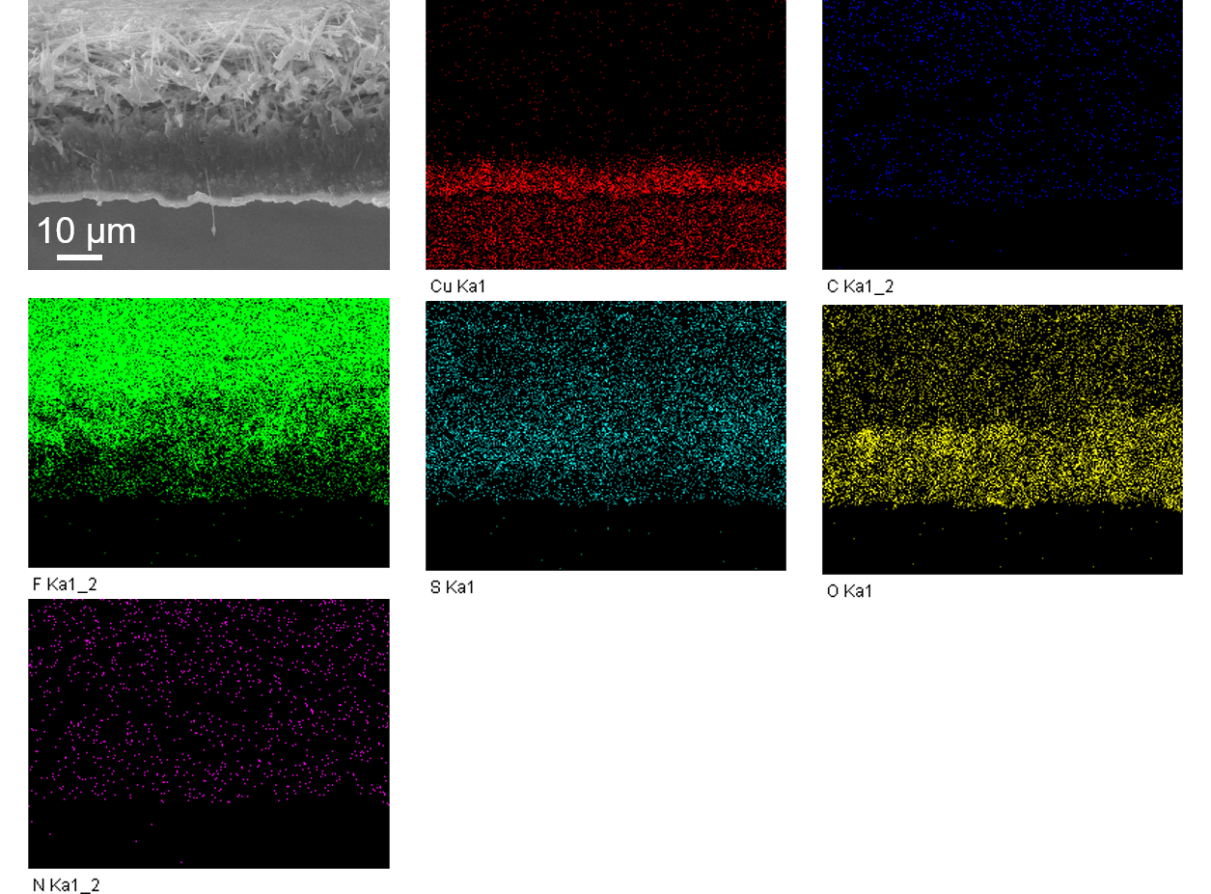
**

**Figure S20**. The cross-section SEM image and EDS mappings of the deposited Li metal in Li//Cu cell with LiF-NFs-IL after discharging 1 h at 1 mA cm^−2^.

**
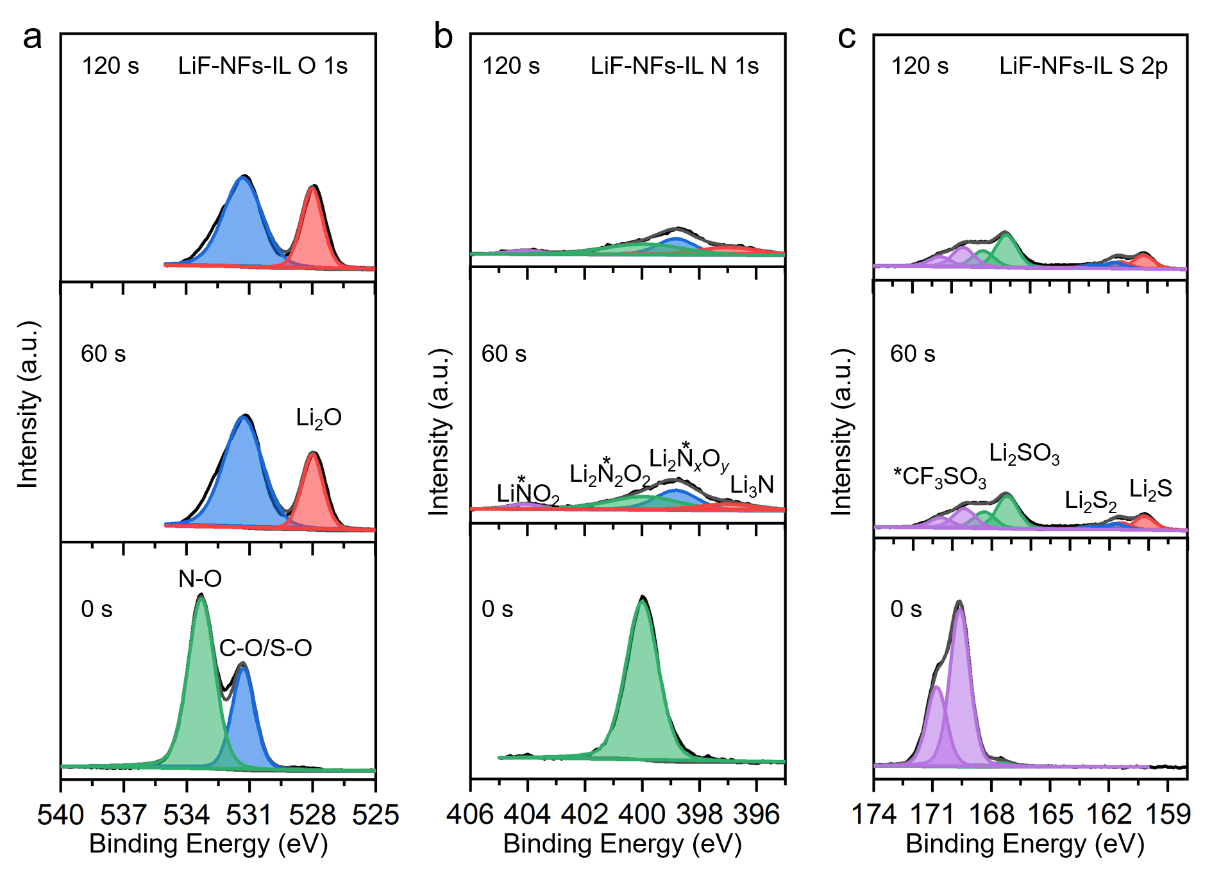
**

**Figure S21**. XPS O 1s (a), N 1s (b) and S 2p (c) depth spectra of SEI film of the deposited Li on Cu foil using LiF-NFs-IL at 1 mA cm^−2^ with 1 mAh cm^−2^.

**
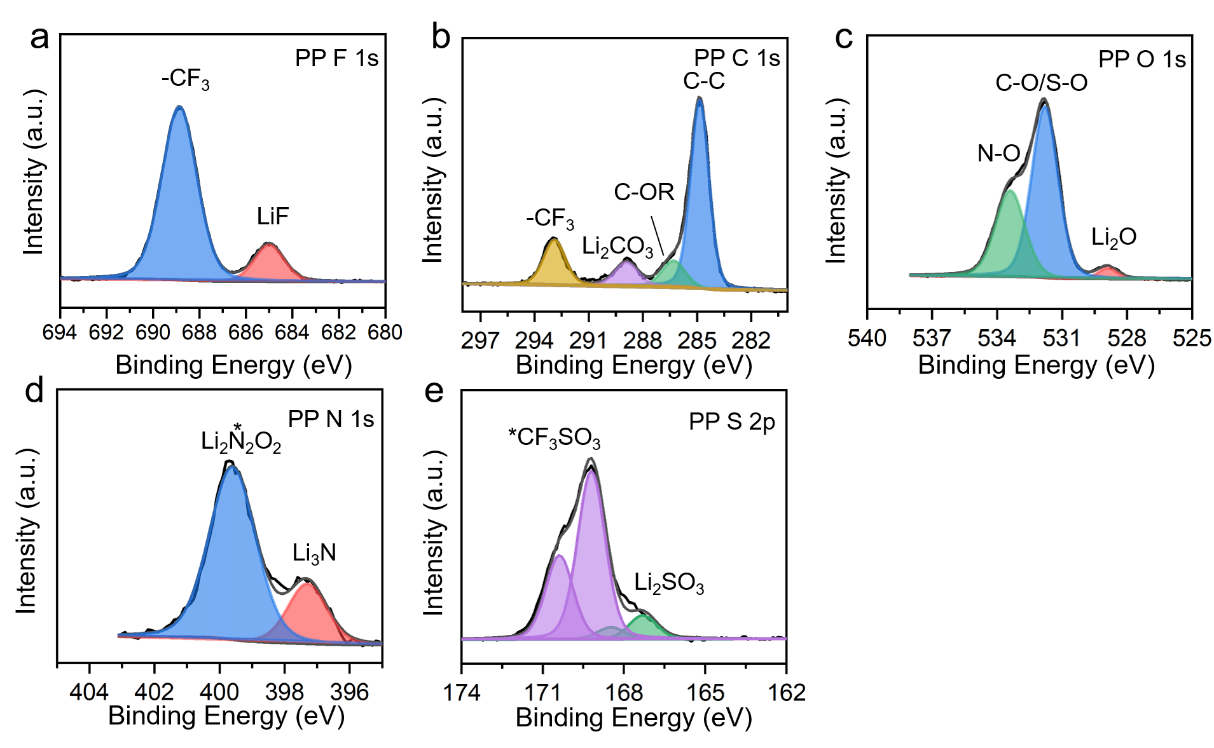
**

**Figure S22**. XPS F 1s (a), C 1s (b), O 1s (c), N1s (d) and S 2p (e) spectra of the deposited Li metal in Li//PP//Cu cell after 10 cycles at 1 mA cm^−2^ with 1 mAh cm^−2^.

**
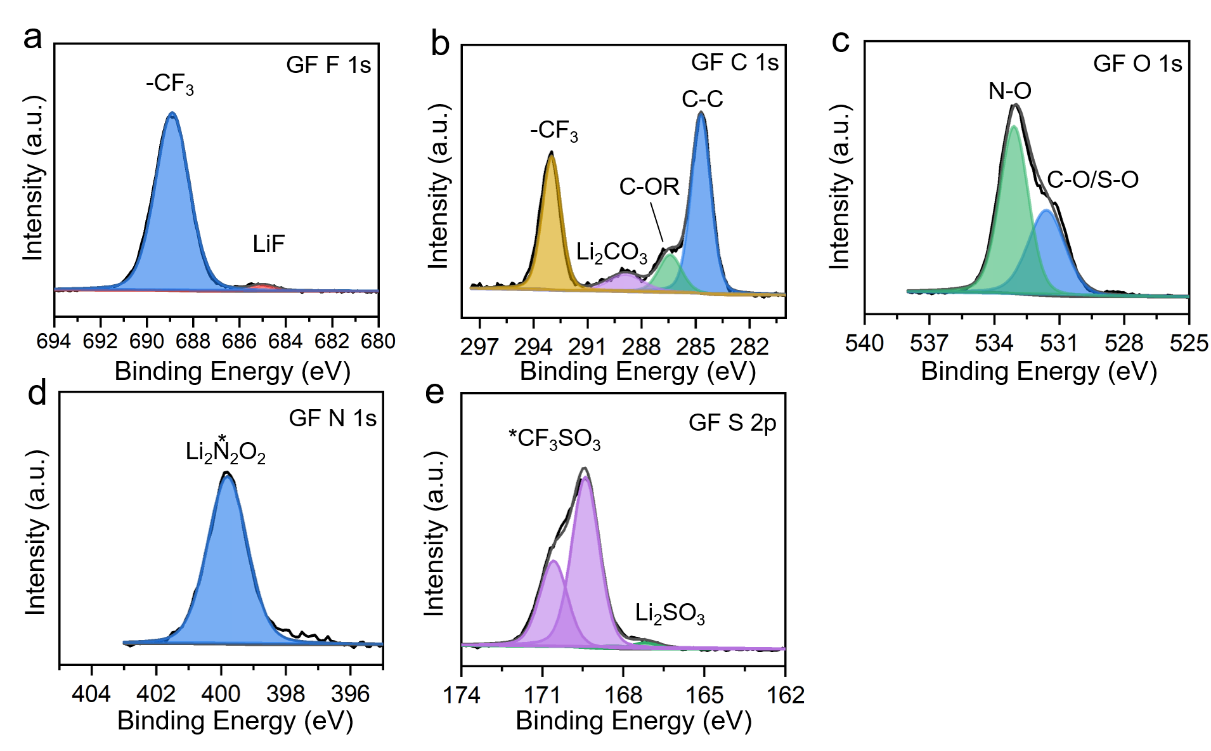
**

**Figure S23**. XPS F 1s (a), C 1s (b), O 1s (c), N1s (d) and S 2p (e) spectra of the deposited Li metal in Li//GF//Cu cell after 10 cycles at 1 mA cm^−2^ with 1 mAh cm^−2^.

**
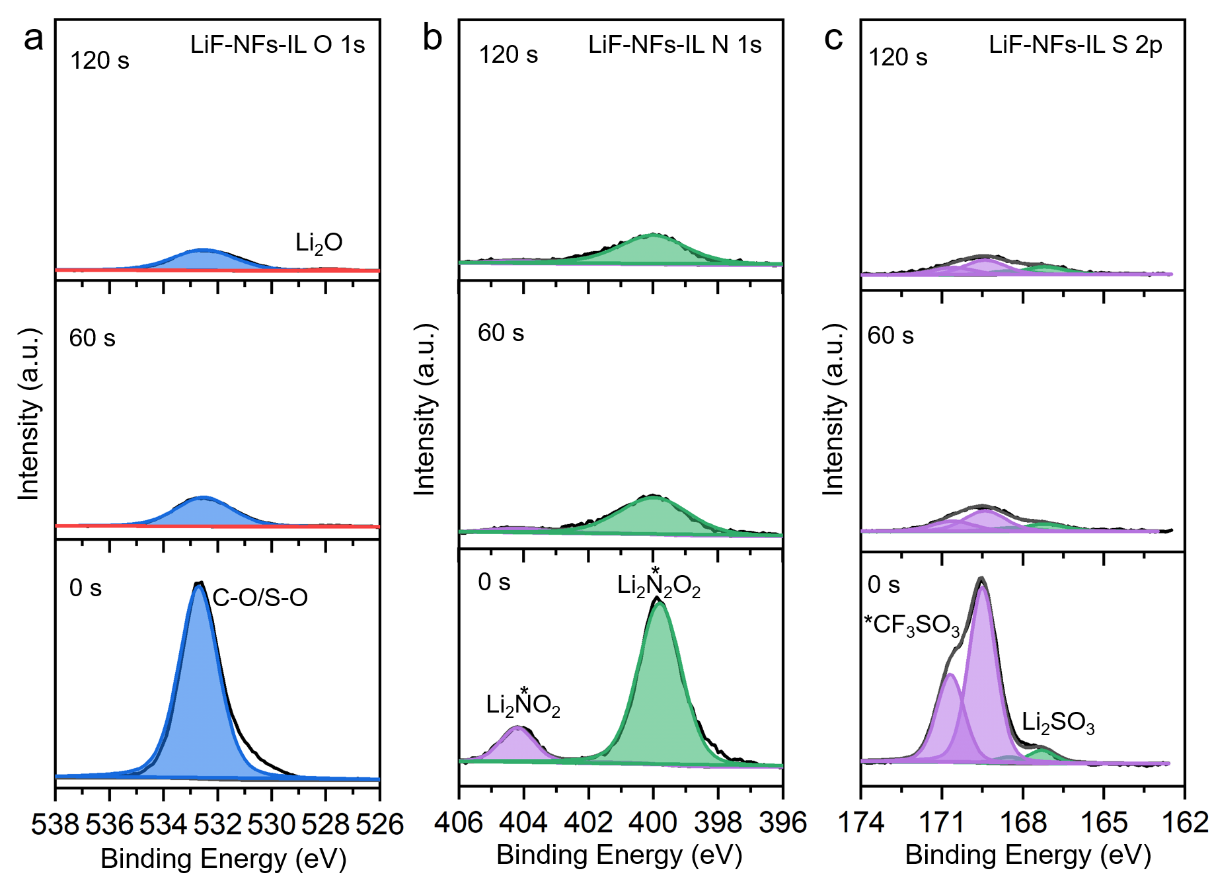
**

**Figure S24**. XPS O 1s (a), N 1s (b) and S 2p (c) depth spectra of the deposited Li metal in Li//Cu cell with LiF-NFs-IL after 10 cycles at 1 mA cm^−2^ with 1 mAh cm^−2^.


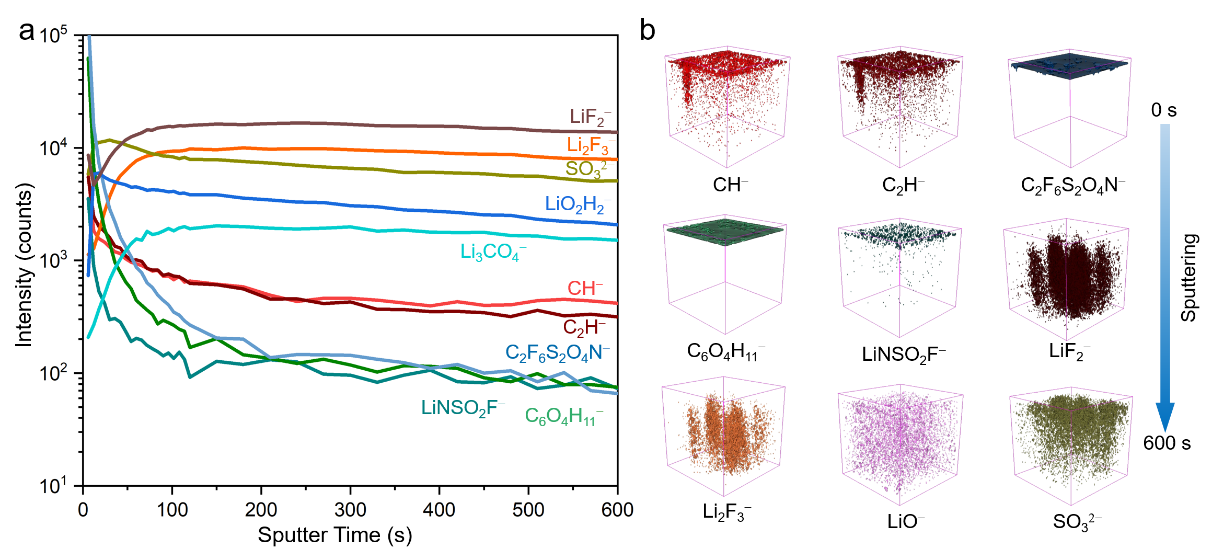


**Figure S25**. TOF-SIMS depth profiles (a) and 3D rendering models (b) of SEI film formed on Li metal in Li//PP//Cu cell after 10th discharge at 1 mA cm^−2^ with 1 mAh cm^−2^.


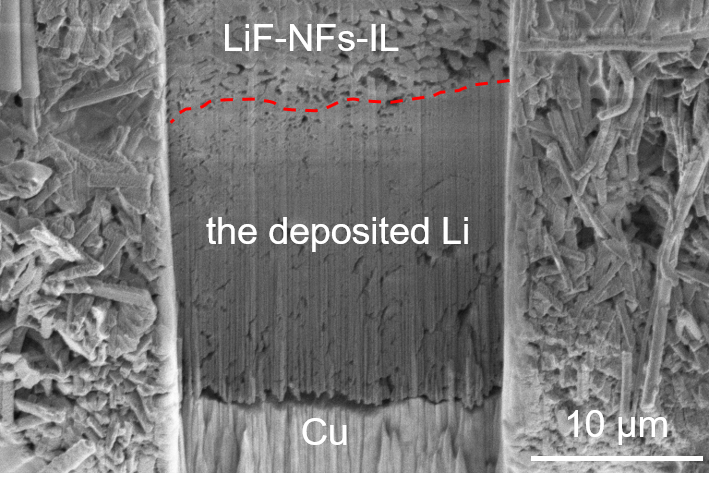


**Figure S26**. FIB-SEM image of the deposited Li metal on Cu foil when used LiF-NFs-IL.

**
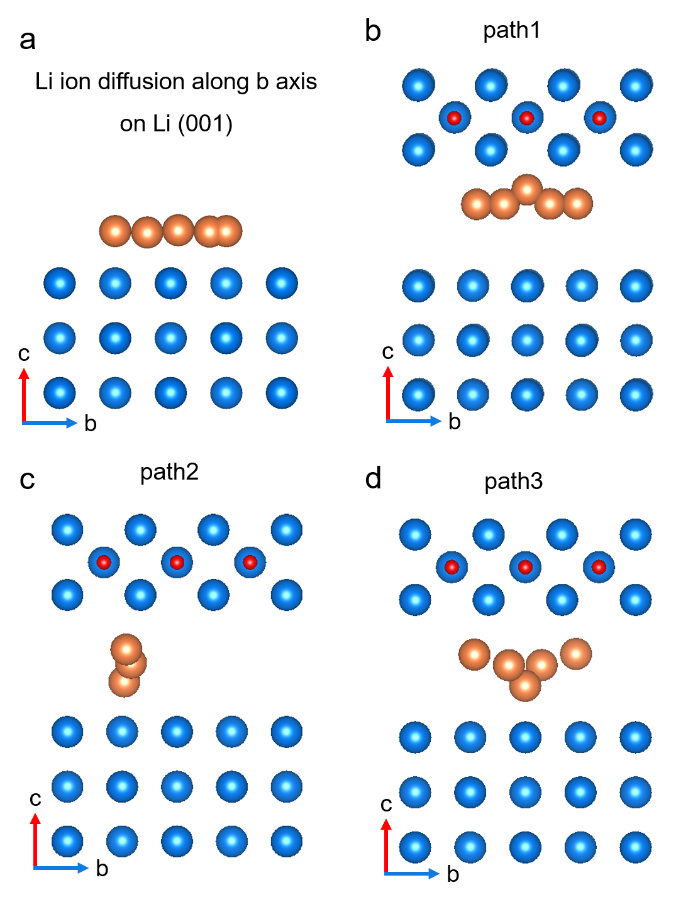
**

**Figure S27**. Diffusion paths of Li atom on Li (001) facet along b axis (a), and in Li-LiF interface along b axis (b), a axis (c) and [110] direction (d).

**
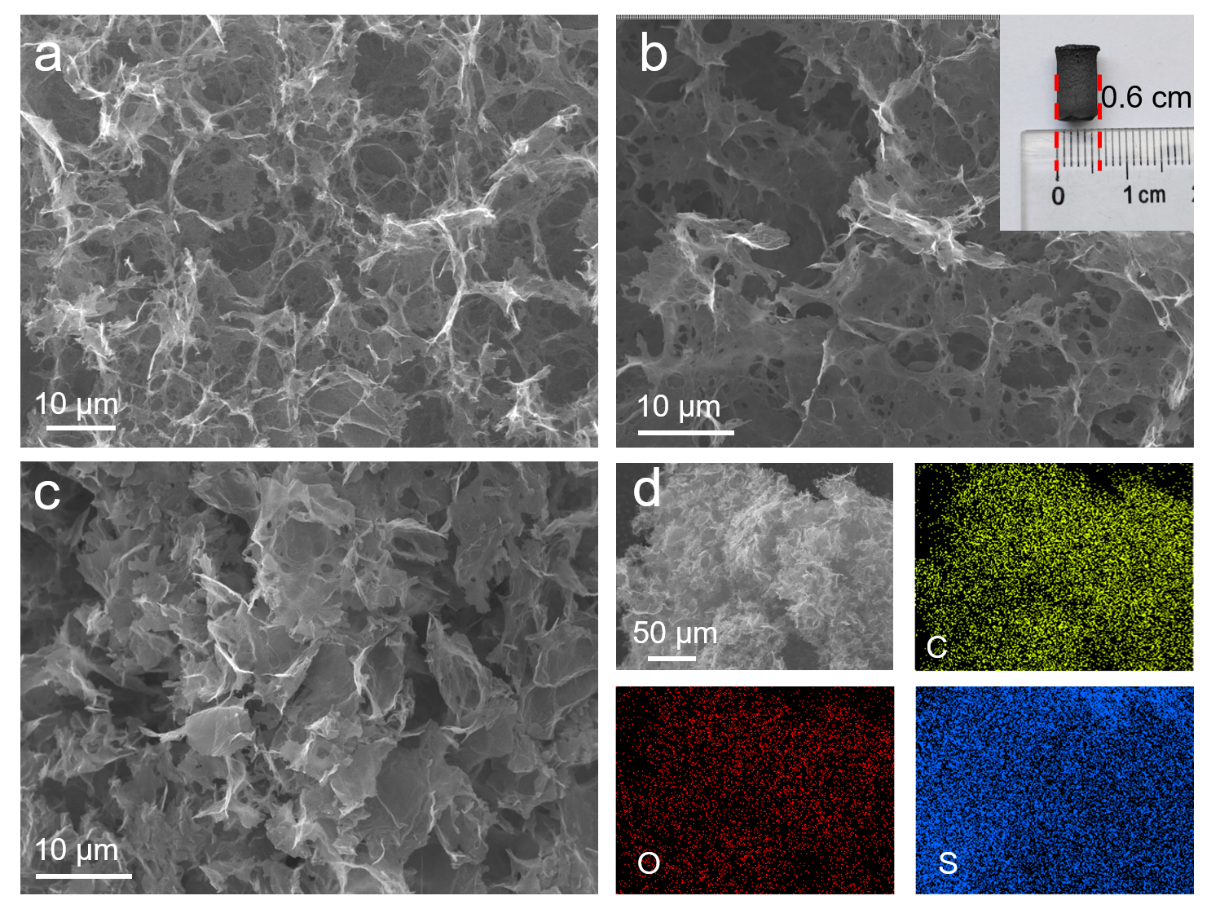
**

**Figure S28**. SEM images of GO xerogel (a), rGO xerogel (b), rGO-S cathode (c). (d) EDS mapping of rGO-S cathode.


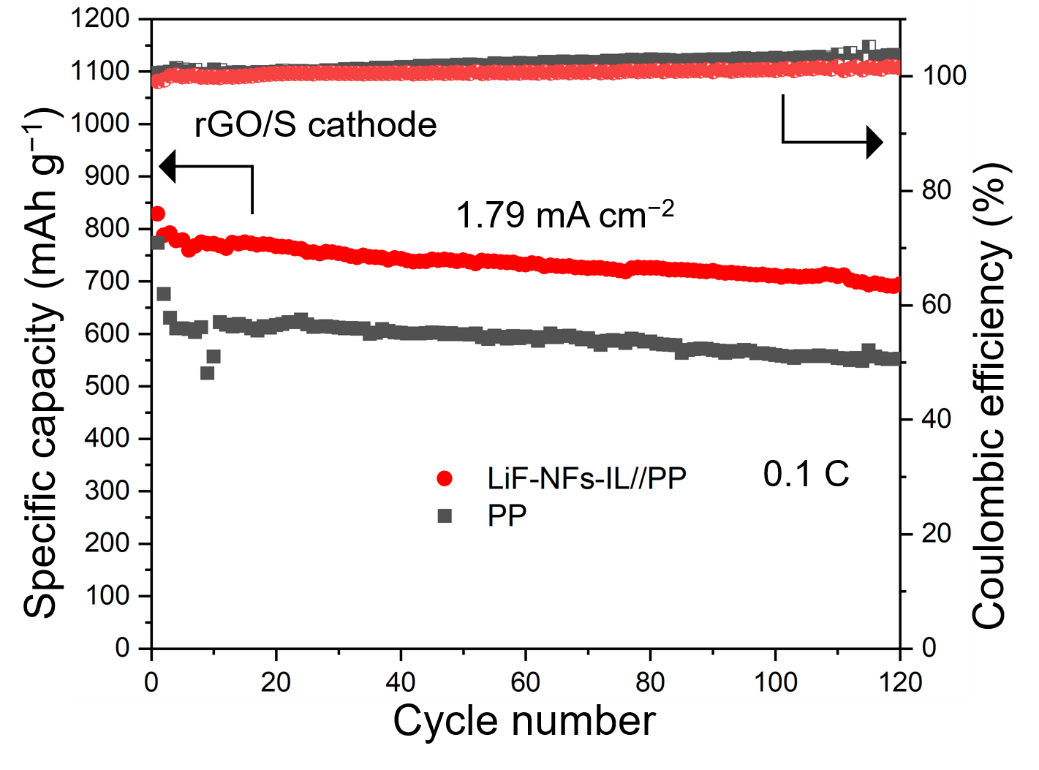


**Figure S29**. Cycling stability of high-loading Li//rGO-S coin cells with and without LiF-NFs-IL at 0.1 C.


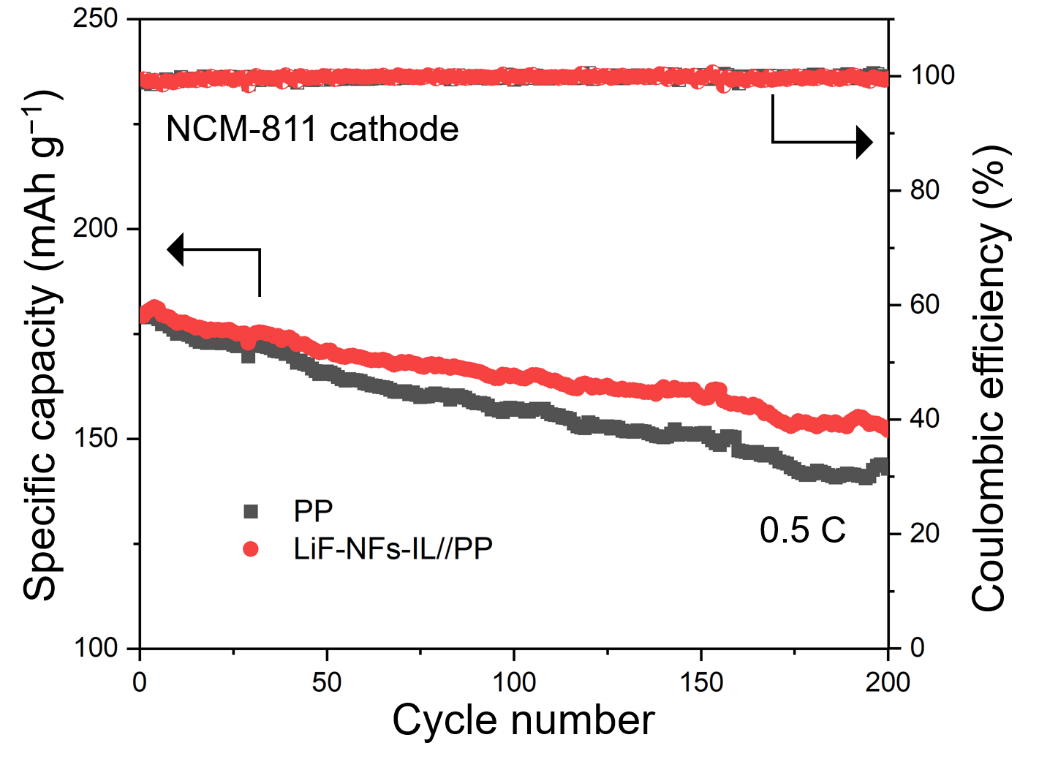


**Figure S30**. Cycling stability of the high-loading Li//NCM-811 coin cells with and without LiF-NFs-IL at 0.5 C.


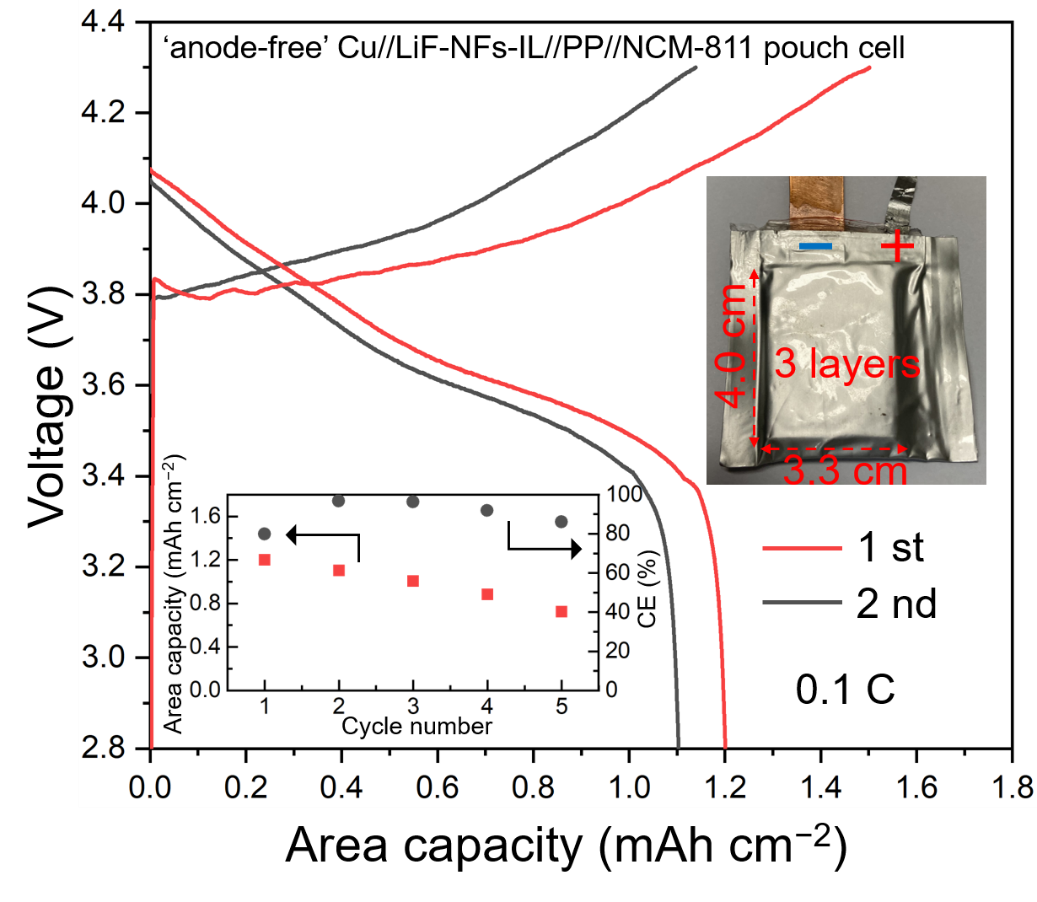


**Figure S31**. Charge-discharge curves of the ‘anode-free’ Cu//LiF-NFs-IL//PP//NCM-811 pouch cell, the insets are the optical photo of pouch cell and its cycling performance.

**
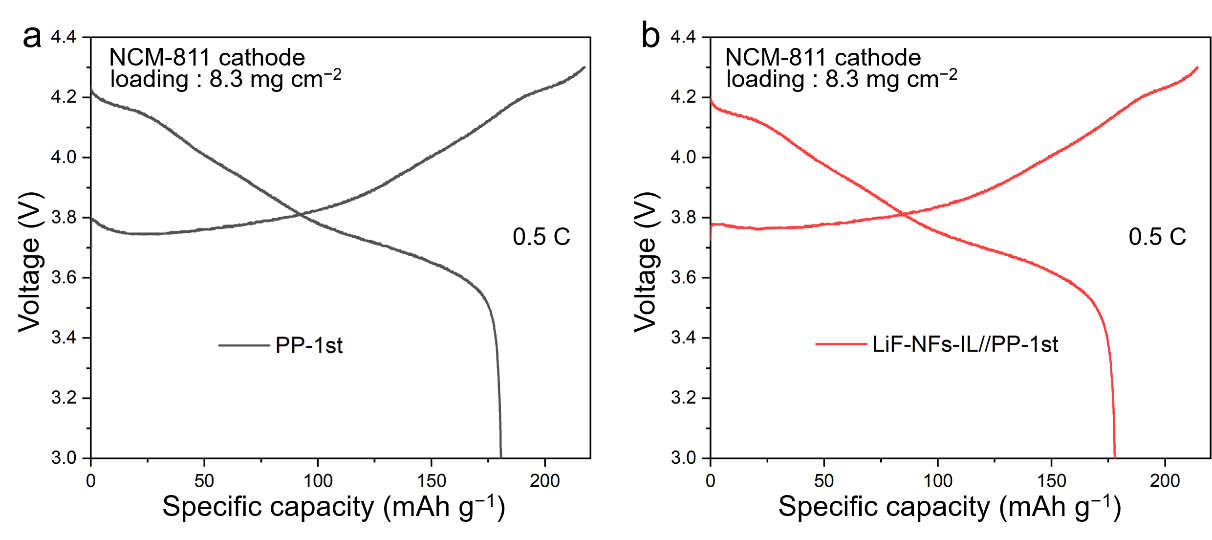
**

**Figure S32**. The first charge-discharge profiles of Li//PP//NCM-811 coin cell (a) and Li//LiF-NFs-IL//PP//NCM-811 coin cell (b).

**Table S1**. A comparison of electrochemical performances in the recent works in relation to the Li metal or separator modifications for high-loading Li-S batteries.

| Modification | Cathode material | Sulfur content in Cathode material | Mass loading (mg cm^−2^) | Area specific capacity /Area current density (mAh cm^−2^/mA cm^−2^) | Cycling  Number (n) | E/S ratio (μL mg_s_ ^−1^) | Ref. |
| --- | --- | --- | --- | --- | --- | --- | --- |
| Our work  LiF-NFs-IL | **rGO-S** | **70 wt%** | **10.7** | **8.86/1.79**  **5.65/17.9** | **120**  **400** | **5** | **-** |
| N-doped porous  carbon nanosheets as current collector | CNT/S | 70 wt% | 10 | 9.84/1.68 | 45 | 10 | [1] |
| LiSPAN@Cu Li metal anode | Se_0.05_S_0.95_PAN | 75 wt% | 10.1 | 12.0/1.0 | 100 | 2.2 | [2] |
| LiPON-coated Li metal anode | KB/S | 70 wt% | 7 | 4.7/0.79 | 120 | 30 | [3] |
| VS_2_-modified PP separator | sulfur powder | 100 wt% | 9.24 | 8.3/3.09 | 120 | 6 | [4] |
| Co and NbC imbedded N-doped porous CF (NbC/Co⊂N-PCFs) as Li host | S @ NbC/Co⊂N-PCFs | 78.5 wt% | 6.7 | 6.1/2.24 | 50 | 10 | [5] |
| 3D CoSe@C as Li host | S/CoSe@C | 70 wt% | 6.2 | 5.8/2.0 | 150 | 4.5 | [6] |
| CoNi nanoparticle-embedded porous conductive scaffold (CoNi@PNCFs) as Li host | S/CoNi@PNCFs | 69.7 wt% | 7.0 | 5.9/2.34 | 50 | 6 | [7] |
| SnF_2_-treated Li anodes | KB/S | 70 wt% | 4.6 | 3.46/1.54 | 100 | 8 | [8] |
| 1,3,5-benzenetrithiol as electrolyte additive | carbon paper/S | - | 4.8 | 5.47/1.8 | 60 | 6 | [9] |
| asymmetric SPOP-Li/LLZNO separator toward the cathode and anode | S/GO | 75 wt% | 6.1 | 5.75/2.04 | 100 | - | [10] |

**References**

[1] Pei F, Fu A and Ye W et al. Robust lithium metal anodes realized by lithiophilic 3D porous current collectors for constructing high-energy lithium–sulfur batteries. *ACS Nano* 2019; **13**: 8337-46.

[2] Jiang Z, Guo H-J and Zeng Z et al. Reconfiguring organosulfur cathode by over-lithiation to enable ultrathick lithium metal anode toward practical lithium–sulfur batteries. *ACS Nano* 2020; **14**: 13784-93.

[3] Wang W, Yue X and Meng J *et al.* Lithium phosphorus oxynitride as an efficient protective layer on lithium metal anodes for advanced lithium-sulfur batteries. *Energy Storage Mater* 2019; **18**: 414-22.

[4] Wang J, Yi S and Liu J *et al.* Suppressing the shuttle effect and dendrite growth in lithium–sulfur batteries. *ACS Nano* 2020; **14**: 9819-31.

[5] Wei Y, Wang B and Zhang Y *et al.* Rational design of multifunctional integrated host configuration with lithiophilicity‐sulfiphilicity toward high‐performance Li–S full batteries. *Adv Funct Mater* 2021; **31**: 2006033.

[6] He J and Manthiram A. 3D CoSe@C aerogel as a host for dendrite‐free lithium‐metal anode and efficient sulfur cathode in Li–S full cells. *Adv Energy Mater* 2020; **10**: 2002654.

[7] He Y, Li M and Zhang Y *et al.* All‐purpose electrode design of flexible conductive scaffold toward high‐performance Li–S batteries. *Adv Funct Mater* 2020; **30**: 2000613.

[8] Guo W, Han Q and Jiao J *et al.* In situ construction of robust biphasic surface layers on lithium metal for lithium–sulfide batteries with long cycle life. *Angew Chem* 2021; **133**: 7343-50.

[9] Guo W, Zhang W and Si Y *et al.* Artificial dual solid-electrolyte interfaces based on in situ organothiol transformation in lithium sulfur battery. *Nat Commun* 2021; **12**: 3031.

[10] Yan W, Yang JL and Xiong X *et al.* Versatile asymmetric separator with dendrite‐free alloy anode enables high‐performance Li–S batteries. *Adv Sci* 2022; doi: 10.1002/advs.202202204.
